# Curation of the *Fasciola hepatica* kinome as a resource for drug target discovery

**DOI:** 10.1186/s12864-025-12513-w

**Published:** 2026-01-16

**Authors:** Sagar Ajmera, Oliver Puckelwaldt, Andreas J. Stroehlein, Simone Haeberlein

**Affiliations:** 1 Institute of Parasitology, BFS, Justus Liebig University, Giessen, Germany; 2 Department for Biological Safety, German Federal Institute for Risk Assessment, Berlin, Germany

**Keywords:** *Fasciola hepatica*, Liver flukes, Helminths, Protein kinases, Kinome, Protein kinase inhibitor, Vandetanib

## Abstract

**Background:**

Fascioliasis is a zoonosis and neglected tropical disease with worldwide distribution and significant economic impact. The search for anthelmintic compounds against *Fasciola hepatica*, one of the parasitic liver flukes responsible for this disease, has become important due to widespread resistances against existing drugs. To facilitate drug discovery, a useful strategy is the identification of proteins that are vital for the parasite. Protein kinases (PKs) emerged as potential targets in several parasites, given their critical role in many biological processes. To date, knowledge of the PKs present in *F. hepatica* is fragmentary.

**Results:**

We curated and classified the kinome of *F. hepatica* using a refined bioinformatics pipeline and found 245 PKs, which represented 2.14% of the parasite’s proteome. Classification of all PKs into their families and sub-families revealed the CMGC group as the largest PK group. A comparison of the kinomes of *F. hepatica* with medically important *Schistosoma* species and the human host revealed key similarities and differences. Based on orthology to human sequences, KEGG functional annotation predicted that 25% of 110 annotated PKs in *F. hepatica* are involved in cancer pathways. We prioritized a panel of related, small-molecule PK inhibitors to assess their efficacy against different *F. hepatica* life stages in vitro. Among these, vandetanib and ruboxistaurin showed lethal effects on immature flukes in vitro at 50 µM concentration, and ruboxistaurin significantly reduced the motility of adult liver flukes.

**Conclusion:**

These findings suggest that repurposing small-molecule PK inhibitors could be a good strategy for obtaining compounds to combat fascioliasis. The newly established *F. hepatica* kinome represents a resource for future target discovery.

**Supplementary Information:**

The online version contains supplementary material available at 10.1186/s12864-025-12513-w.

## Background

The parasitic flatworm *Fasciola hepatica*, also known as the common liver fluke, infects a wide spectrum of host animals as well as humans [1]. The disease fascioliasis is a food-borne infection that causes tremendous economic losses in livestock industry, exceeding 3 billion dollars per year [2]. WHO classifies fascioliasis as a neglected tropical disease (NTD) [3]. The parasite’s life cycle is complex and develops via multiple life stages in intermediate and final hosts. In the mammalian final host, liver flukes affect the liver and cause symptoms including abdominal pain, nausea as well as liver dysfunction. The widespread presence of *F. hepatica* raises food safety concerns worldwide [4].

The current strategies against fascioliasis for both humans and animals crucially depend on the drug triclabendazole (TCBZ). However, its intensive use has led to the emergence of TCBZ-resistant strains of *F. hepatica*, which have been documented in several countries [5–8]. Additionally, in the absence of a vaccine, main control strategies focus on the exploration of new therapeutic options. Protein kinases (PKs) have emerged as promising drug targets in parasites causing NTDs, such as cestodes [9], different schistosome species [10–12] and various protozoans [13, 14]. The availability of kinome data for these and other parasites represents a resource for the identification of proteins with vital function for a pathogen, for prioritizing genes for functional characterization, and ultimately, the discovery of novel therapeutic targets.

PKs are key regulators of various cellular processes including cell proliferation, differentiation, apoptosis, migration, and metabolism. In humans, the kinome covers 518 PKs [15]. Among the proteins in focus as prime targets for drug development, PKs represent the second largest group after G-protein coupled receptors [16]. Given that PKs are important players in cancer development, numerous FDA-approved, small-molecule PK inhibitors serve as anti-cancer agents. Until now, the list of such inhibitors has extended to 94 compounds and many more are undergoing clinical trials (http://www.icoa.fr/pkidb) [17, 18]. Building upon a concept of repurposing existing PK inhibitory drugs for the treatment of schistosomiasis, introduced by Dissous and Grevelding [19], this approach could deliver novel therapeutic approaches also against fascioliasis. This is further underlined by our previous work where in vitro activity of imatinib (a tyrosine kinase inhibitor) against all pathogenic life stages of *F. hepatica* was found, as well as a high transcriptional expression of the presumed target ABL1 in those stages [20, 21].

The aim of this study was to provide a curated dataset of the *F. hepatica* kinome. This data was compared to kinomes of related parasitic flatworms and of the human host to reveal conserved and potential parasite-specific PKs. Finally, PK inhibitors were prioritized and screened in vitro to assess their effects on the vitality of *F. hepatica*.

## Methods

### Ethical statements

Animal experiments using rats (*Rattus norvegicus*) as model hosts were performed in accordance with Directive 2010/63/EU on the protection of animals used for scientific purposes and the German Animal Welfare Act. The experiments were approved by the Regional Council (Regierungspraesidium) Giessen (V54-19c20 15 h 02 GI 18/10 Nr. A16/2018).

### Prediction and curation of the *F. hepatica* kinome

**Fig. 1:**
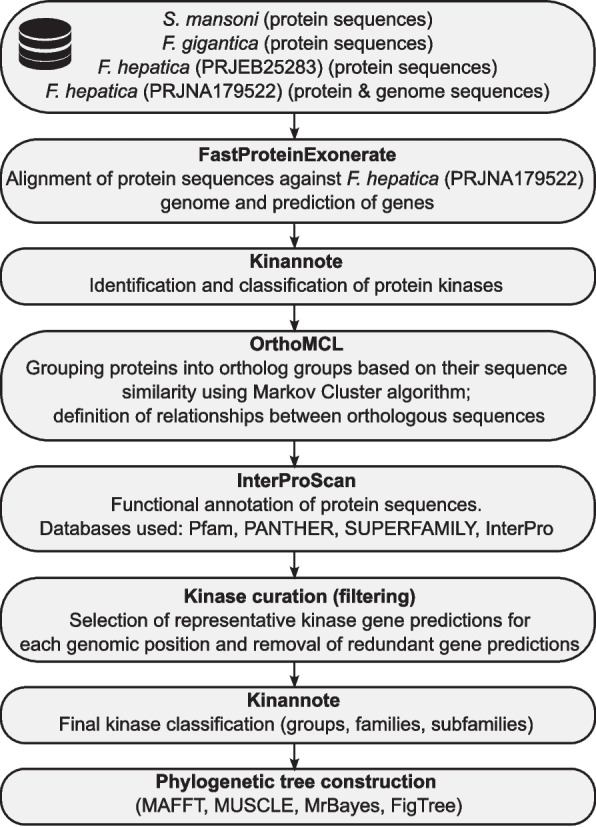
Workflow for the prediction and curation of the *F. hepatica* kinome

A bioinformatic pipeline previously established for a schistosome kinome analysis [11] was modified (Fig. 1) and used for predicting, curating, and defining PKs in the *F. hepatica* genome (PRJNA179522, annotation version 2021–12-WormBase) published on WormBase ParaSite vWBPS18 (https://parasite.wormbase.org/index.html) [22]. Firstly, we employed the Kinannote program [23] on the genome using the metazoan (-m) option, which generated a draft kinome and classified PKs into groups, families, and subfamilies. We predicted the genes using two closely related trematode species and one additional *F. hepatica* genome. We used the inferred proteomes of *S. mansoni* (PRJEA36577), *F. gigantica* (PRJNA230515), and *F. hepatica* (PRJEB25283) to complement existing gene models in the *F. hepatica* (PRJNA179522) genome (all obtained from WormBase ParaSite vWBPS18) employing the FastProteinExonerate script (https://github.com/stroehleina/FastProteinExonerate). FastProteinExonerate uses pBLAT [24], bedtools [25], exonerate [26] and gffread [27]. We then used OrthoMCL [28, 29] to group proteins into orthologous groups and define the relationship between sequences. Functional annotation was carried out using InterProScan v5.63–95.0 [30], which provided information about PK domains using the Pfam v36.0 [31], PANTHER v16 [32], and SUPERFAMILY v2 [33] databases. With all the above information, we then reduced redundant and overlapping gene models to single representative gene loci using R v4.3.0 [34] employing the tidyverse [35] package. For newly predicted genes, numeric identifiers were created using the prefix “FhPK” in the final dataset; for reference, the original identifiers—as they are found in the WormBase ParaSite database—were kept in Supplementary data 1.

### Phylogenetic tree construction

Phylogenetic trees for the individual classified PK groups of *F. hepatica* and the orthologous kinases being part of the already published kinome of *S. mansoni* [12] excluding the genes with unclear PK class identity (unclassified) were constructed. First, multiple sequence alignments of PK sequences were generated with MAFFT v7.520 [36] using the most accurate alignment option (L-INS-i; parameters *-local -maxiterate* 1000). The alignments from MAFFT were further optimized using MUSCLE v5 [37] using the refine option (*-refine*). The refined sequences were subjected to MrBayes v3.2.7a [38] to perform Bayesian analysis, where posterior probabilities were calculated using the LG + I + G (Le and Gascuel + invariable sites + gamma distributed rate heterogeneity) model with fixed state frequencies. In total, 1,000,000 trees were generated, from which 25% trees were discarded as burn-in. The remaining 75% trees were used to construct the majority-rule consensus tree resulting from the analysis. The program FigTree v1.4.4 (https://github.com/rambaut/figtree/releases) was used to visualize and export the trees as figures. The percentage posterior probability values of all trees are listed in Supplementary Data 2.

### KEGG functional annotation

Functional annotation with respect to metabolic pathways associated with PKs was achieved using the default settings at the BlastKOALA webserver [39] (https://www.kegg.jp/blastkoala/).

### Kinase sequence comparisons

A comparative analysis was performed between the kinases of *F. hepatica* and the kinomes of *S. mansoni* [12, 40]*, S. haematobium* [11]*,* and *H. sapiens* [15]. The protein sequences were searched against the Pfam-A database [31] using HMMER v3.3.2 [41]. Domain sequences matching the Pfam entry for “protein kinase domain” (PF00069) were extracted. Then, protein sequences having fragmented domains (two or more domain sequences shorter than 140 amino acids) were merged. To compare the sequences, we grouped the sequences using Orthofinder [42]. Next, we performed groupwise all vs all global alignments using EMBOSS needle v 6.6.0.0 [43]. Finally, for each *F. hepatica* kinase, the best alignment (based on percent identity) was considered. For the atypical kinases, comparisons were performed using full-length sequences via local BLAST [44] as they lack sequence similarity to Hidden Markov Models (HMM) protein kinase profiles [45].

### Maintaining and harvesting *F. hepatica*

Metacercariae of an Italian *F. hepatica* strain were purchased from Ridgeway Research (UK) and used to infect five week-old male Wistar rats (RjHan: WI, Janvier, France). Oral infection was done with 25 metacercariae to obtain immature flukes and 20 metacercariae for adult flukes. Immature worms were harvested at four weeks post infection (p.i.) from livers, and adult worms at 12–15 weeks p.i. from bile ducts. Gathered *F. hepatica* were incubated in RPMI-1640 with 5% chicken serum and 1% ABAM (10,000 units of penicillin, 10 mg streptomycin, and 25 mg amphotericin B per ml) (all purchased from Gibco) at 37 °C with 5% CO_2_ overnight to allow clearance of gut contents. Worms were then further subjected to PK inhibitor treatment.

### In vitro evaluation of PK inhibitors

The following commercially available compounds were tested on the flukes: vandetanib (ZD6474), foretinib (XL880), tyrosine-kinase-IN-1 (XL999), ruboxistaurin (LY333531), and triclabendazole (TCBZ) (all compounds from Selleck Chemicals). Stock solutions of 10 mM were prepared in dimethyl sulfoxide (DMSO). Different concentrations of the PK inhibitors (25 µM, 50 µM, and 100 µM) were used to evaluate anthelmintic activities on the flukes. The same volume of the solvent DMSO served as negative control. The worms were cultured in RPMI-1640 containing 5% chicken serum and 1% ABAM at 37 °C for three days, with exchange of media, DMSO and PK inhibitors every 24 h. Inhibitor-induced effects on worm viability were assessed every 24 h using a stereo microscope at 10 × magnification (M125 C, Leica, Germany). Worm motility was scored from 0 to 3 as described previously [20] (0 = no movement even after mechanical stimulation with forceps, 1 = minimal movement, 2 = reduced movement, 3 = normal movement). Representative videos for each score can be found in Supplementary videos 1–8. The mean motility score was calculated for flukes (n = 4) acquired in three different independent experiments.

### Statistical analyses

Statistical analyses were performed in R v4.3.0 [34]. A non-parametric analysis of variance was performed using a Wilcoxon rank sum test. *P*-values < 0.05 were considered as statistically significant. All associated data can be found in the Supplementary data 2.

## Results

### Predicting and defining the kinome of *F. hepatica*

A first draft kinome of *F. hepatica* was generated using Kinnanote. This covered 212 PKs, out of which 200 eukaryotic protein kinases (ePKs) were classified into different PK groups, families, and subfamilies. Four PKs were classified as atypical PKs while the remaining eight PKs remained unclassified. We then adopted a bioinformatics pipeline [11] (Fig. 1) to predict, identify and curate the PK sequences. Through this iterative pipeline and manual curation, we were able to incorporate 29 additional PKs, expanding the dataset to 241 ePKs and 4 aPKs ensuring a more comprehensive representation of the *F. hepatica* kinome. This refined kinome with a total of 245 PKs (among which 233 classified) represents 2.15% of the 11,217 protein coding genes within the genome of *F. hepatica*.

**Fig. 2:**
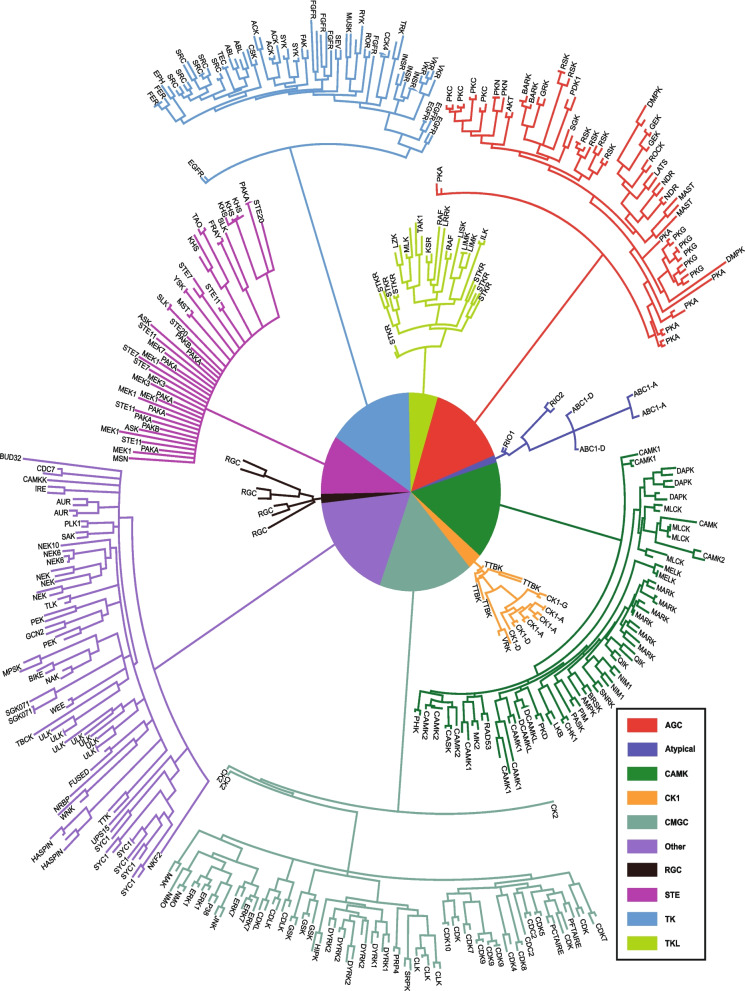
Phylogenetic analysis of protein kinases (PKs) of *Fasciola hepatica* and *Schistosoma mansoni*. Protein kinase groups are indicated by color. Detailed phylogenetic trees including posterior probabilities with comprehensive nodal support values (providing statistical support for the inferred relationships) are provided as high-resolution images in Supplementary data 2, a list of the gene identifiers is provided in Supplementary data 4

The identified ePKs belong to the nine known major groups of PKs, which are classified based on their catalytic domain (AGC, CAMK, CK1, CMGC, RGC, STE, TK, TKL, and Others) and are further clustered into families and subfamilies based on accessory domains and sequence similarity (Table 1 and Supplementary data 1/Table S1). For 31 PKs, family classification could not be delivered by Kinnanote. For 19 of these, classification was subsequently achieved by pairwise alignment of *F. hepatica* and *S. mansoni* PK sequences available from the published kinome (Supplementary data 1/Table S2) [12]. A phylogenetic tree was constructed from these data, which includes all 233 classified PKs for *F. hepatica* and 263 PKs of *S. mansoni* (Fig. 2, Supplementary data 2 and 4). The previously unassigned PKs belonged to (sub)families of the AGC group (2 PKs), CAMK group (5 PKs), and TK group (12 PKs) (Supplementary data 1/Table S2).

**Table 1:** Summary of the *F. hepatica* kinome, including initial draft classification based on Kinnanote and the final curated classifications. The number of protein kinases (PKs) per PK group is shown

PK group	Draft kinome	Curated kinome
AGC	26	34
CAMK	35	38
CK1	5	8
CMGC	41	43
Other	30	35
RGC	4	4
STE	18	20
STK (unclassifiable)	8	12
TK	29	33
TKL	12	14
atypical/aPKs	4	4
Total	212	245

*PK* Protein kinases, *AGC* PK A, G, and C families, *CK1* casein kinase 1, *CMGC* including cyclin-dependent kinases (CDKs), mitogen-activated protein kinases (MAP kinases), glycogen synthase kinases (GSK) and CDK-like kinases, *CAMK* calcium- and calmodulin-regulated kinases, *RGC* receptor guanylate cyclase, *STE* homologs of the yeast STE7, STE11 and STE20 genes, *TK* tyrosine kinases, *TKL* tyrosine kinase-like kinases, *STK* serine/threonine protein kinases

### Eukaryotic protein kinases of *F. hepatica*

Members of the ePK family share a region of approximately 250 amino acids in length that forms the catalytic domain and distinguishes ePKs from other kinases [46]. The five largest groups of ePKs in *F. hepatica*, each covering more than 10% of the total number of ePKs, were CMGC, CAMK, AGC, TK and Others (Supplementary data 1/Table S1). The largest PK group CMGC (*n* = 43) represents 17.84% of all ePKs in the *F. hepatica* kinome. CMGC kinases typically play important roles in cellular signaling pathways, including proliferation, differentiation and apoptosis [47]. One of the most well-known group members are cyclin-dependent kinases (CDKs), which regulate cell-cycle progression, and mitogen-activated protein kinases (MAPKs), which in mammals comprise three major groups: extracellular signal-related kinases (ERKs). These are typically stimulated by growth related signals, c-Jun N-terminal kinases (JNK) and p38, which both are activated by stress stimuli [47]. In *F. hepatica*, we defined the following family members within the CMGC group: 16 CDKs, eight dual specificity tyrosine-phosphorylation-regulated kinases (DYRK2), seven MAPKs (including one JNK, one p38, one NMO and four ERK kinases), three cyclin-dependent kinases-like kinases (CDKL), three glycogen synthase kinases-3 (GSK), two casein kinase II subunit (CK II), two dual specificity protein kinases (CLK), one male germ-associated kinase (MAK), and one serine-arginine protein kinase (SRPK).

The second largest PK group with 38 members (15.77%) covers calcium- and calmodulin-dependent kinases (CAMK). In helminths, CAMKs play essential roles in numerous cellular processes, ranging from muscle contraction to nerve impulse transmission [48]. In *F. hepatica*, we identified nine kinases representing CAMK1 and CAMK2 family members, 16 calcium- and calmodulin-regulated kinase-like (CAMKL) members, three death-associated protein kinases (DAPK), three myosin light-chain kinases (MLCK), and one member of each of the families calcium/calmodulin-dependent serine protein kinase (CASK), doublecortin-like and CAM kinase-like (DCAMKL), MAP kinase-associated protein kinase (MAPKAPK), Pim-1 proto-oncogene (PIM), phosphorylase kinase (PHK), protein kinase D (PKD), and checkpoint kinase 2 (RAD53). PKs belonging to the group “Others” display sequence characteristics that differ from the other eight main ePK groups. With 35 members, this group represents 14.52% of the ePKs and includes five SCY1-like protein 1 (SCY1) members, five Unc-51 like kinases (ULK), four NIMA related kinases (NEK), four pancreatic eukaryotic translation initiation factor 2 alpha kinase 3 (PEK) members, three numb-associated kinases (NAK), two polo-like kinases (PLK), two aurora kinases (AUR), and one member each for another ten families.

The AGC group covers Ser/Thr protein kinases and is named after three representative families, protein kinase A, G and C [49]. In *F. hepatica*, the AGC group covers 34 kinases (14.11%) belonging to 13 families: five protein kinases G (PKG), five ribosomal protein S6 kinases (RSK), five dystrophy myotonic protein kinases (DMPK), five protein kinases A (PKA), four protein kinases C (PKC), three G-coupled receptor kinases (GRK), two nuclear DBF2-related kinases (NDR), one protein kinase B (AKT), and four other families with one member each. Among these PKs, two were initially unclassifiable (D915_002893 and FhPK_0023) by Kinannote, but were annotated as DMPK and PKA, respectively, after phylogenetic tree analysis (“Missing genes” in Supplementary data 1/Table S2).

The tyrosine kinase group TK (*n* = 33, 13.69%) can be functionally separated into receptor tyrosine kinases (RTKs) and non-receptor TKs (cytoplasmic TKs, CTKs). In metazoans, phosphorylation of tyrosine residues is a key regulator of signal transduction related to processes such as cell differentiation, organ development, and tissue homeostasis. RTKs are known to respond to external signals, such as growth factors, and propagate them within the cell by phosphorylating internal targets [50, 51]. In *F. hepatica*, 18 RTKs were annotated: four fibroblast growth factor receptors (FGFR), three members of the epidermal growth factor receptor (EGFR) family, three insulin receptors (INSR), and one member each being part of nine other families. Another 15 TK group members were classified into CTKs with: five Src family kinases (SRC), two abelson murine leukemia homologs (ABL), two activated Cdc42-associated tyrosine kinases (ACK), two spleen associated tyrosine kinases (SYK), as well as one family member each representing a C-terminal SRC kinase (CSK), focal adhesion kinase (FAK), feline encephalitis virus-related kinase (FER), and a tec protein tyrosine kinase (TEC). Of these genes, twelve were not assigned to a family by Kinannote, but could be classified by phylogenetic tree analysis, e.g. FhPK_0008 as venus kinase receptor (VKR) and D915_003619 as FGFR (see “Missing genes” in Supplementary data 1/Table S2).

Each of the remaining four kinase groups—STE, TKL, CK1 and RGC—covered less than 10% of the total ePK number of *F. hepatica*. The STE group contributed to 8.30% of ePKs with 20 members. This group contained several members involved in MAP kinase cascades, in which one kinase phosphorylates another kinase. We found four MAP kinase kinases (MAP2K) homologous to yeast Ste 7 (STE7), three MAP kinase kinase kinases (MAP3K) homologous to yeast Ste 11 (STE11), and 13 MAP kinase kinase kinase kinases (MAP4K) homologous to yeast Ste 20 (STE20). The sixth PK group covers tyrosine kinase-like (TKL) kinases (*n* = 14, 5.81%), named for their close sequence similarity to TKs. Here, among others, seven serine threonine kinase receptors (STKR) and four mixed lineage kinases (MLK) were found. A generally small ePK group of metazoans is casein kinase 1 group (CK1), which covered just eight PKs in *F. hepatica*, representing 3.32% of ePKs: five CK kinases (CK1-A, D & G), two tau tubulin kinases (TTBK), and one vaccinia-related kinase (VRK). Finally, the smallest group of ePK covered just four members belonging to the receptor guanylate cyclases (RGC, 1.66%).

### Atypical and unclassified protein kinases of *F. hepatica*

In addition to ePKs, four atypical PKs (aPKs) were identified (Supplementary Data 1/Tables S1, S4). Atypical PKs share a spatially conserved kinase fold but do not share sequence similarity to ePKs. Four atypical kinase families have proven protein kinase activity and are considered as *bona fide* aPKs, which are Alpha, PIKK, PDHK, and RIO. Among these, we only found two RIO kinases (RIOK-1 & 2) in the *F. hepatica* kinome. The other two aPKs represent ABC1-domain containing kinases (ABC1- A & D), although experimental proof of kinase activity in the ABC1 kinase protein family is under debate [15, 46].

Furthermore, Kinannote classified 12 more genes as serine/threonine PKs (STK): FhPK_0019, FhPK_0028, FhPK_0032, D915_002076, D915_003160, D915_004132, D915_004196, D915_006252, D915_006916, D915_007212, D915_008949, and D915_009626. These genes scored below the cut-off HMM value for the classification into PK groups or families and remained therefore unclassified, but were retained as a part of the final kinome (Supplementary Data 1/Table S1). The domain architecture for these unclassifiable STK genes was validated via the SMART webserver [52] (https://smart.embl.de), which displayed a PK catalytic domain with unclassified specificity for all 12 genes. Lastly, six genes were defined as ‘twilight’ hits by Kinannote [23], i.e., PK subdomain-containing proteins that might be distant homologs of ePKs (FhPK_0010, D915_001945, D915_003040, D915_003717, D915_005953, and D915_007708). These twilight hits were not considered as part of the final kinome.

### Kinome comparison across trematode species and differences to the human kinome

**Fig. 3:**
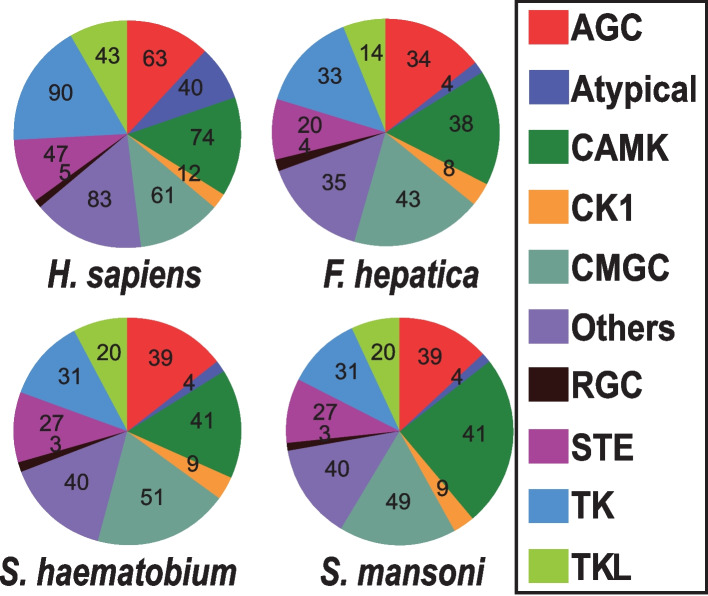
Comparison of human and trematode kinomes. Pie charts illustrating the ten PK groups within the kinomes of *F. hepatica*, *S. mansoni*, *S. haematobium*, and *H. sapiens.* The number of kinases per group is indicated

We compared the kinome of *F. hepatica* with existing kinome datasets of two other trematode species, *S. mansoni* [12, 40] and *S. haematobium* [11], to gain insight into evolutionary relationships and functional similarities within this class of parasites. We also included the human kinome [15] to enable identification of potentially trematode-specific PKs. The three trematode kinomes comprise 233, 263 and 265 classified PKs for *F. hepatica*, *S. mansoni* and *S. haematobium*, respectively, while the human kinome involves 518 PKs. A group-level analysis of the four kinomes delivered the number of PKs found for the nine ePK groups and the aPK group of each species (Fig. 3). PK group sizes were largely comparable among all three trematode species, with CAMK and CMGC as the largest groups. However, group sizes in the human kinome clearly differed from the trematodes. In humans, the TK and Others groups (18% and 16%) comprise larger and the CMGC group (12%) smaller fractions within the kinome compared to the same groups within the trematode kinomes (for CMGC: 18% for *Fh*, 19% for *Sm* and 19% for *Sh*). In addition, the total number of PKs in humans is significantly higher than in trematodes across all PK groups, reflecting the greater complexity of the human kinome.

A global pairwise comparison of the kinase domain sequences revealed 67 PKs in *F. hepatica* for which either no human homolog could be found or for which the identity of human PK domain sequences was very low (< 30%), and thus an assignment as homolog was not possible with certainty (Table 2). The majority of these *Fasciola* genes had homologs in one or both schistosome species. In the TK group, as example, VKR genes (Smp_153500, Smp_019790, ShVKR2, ShRTK, D915_003706 and FhPK_0008) were represented only in the three parasite kinomes, with no homologs for *H. sapiens*. When considering the full-length sequences, human insulin receptors appeared as closest homologs (Supplementary data1/Table S11) [53–55].

**Table 2:** Summary of *F. hepatica* PKs with PK domain sequence identity < 30% to the best human PK hit

PK group	*Fh*_Identifier	*Hs*_Identifier	% Sequence identity	% Sequence similarity
Others	D915_009688	SCYL3	8.5	13.1
Others	D915_007882	PRPK	13.5	18.7
AGC	D915_002893	LATS1	14.7	21
AGC	D915_008016	LATS1	16.2	19.3
AGC	FhPK_0023	PKG2	17.8	25.9
Others	FhPK_0018	CaMKK1	18.9	28.7
TKL	D915_003381	ALK2	20.5	29.3
Others	D915_007720	NRBP1	20.5	32.4
Others	D915_004111	CDC7	22.5	27.8
CAMK	D915_007740	CaMK4	22.7	40.5
TKL	D915_009735	ALK7	22.9	34.3
RGC	D915_001650	ANPb	24.2	38.9
STE	D915_002287	MAP3K1	24.6	35.6
CAMK	D915_001717	LKB1	24.6	35.7
TK	D915_008445	FRK	25.6	37
Others	D915_004720	SgK071	26.5	45.9
Others	D915_008234	Haspin	26.8	39.3
TK	D915_001277	LCK	27	40.3
Others	D915_005804	IRE2	27.1	36.7
TKL	D915_010455	ALK7	28	39.1
CAMK	D915_005826	DRAK1	28.7	45.3
TK	D915_006371	RYK	28.9	42.2
Others	D915_006868	Haspin	29.6	42.2

When comparing the ePKs of the three trematodes among each other, four kinases were only represented in the *F. hepatica* kinome, without homologs in any of the schistosome species based on the comparison of PK domains (Supplementary data 1/Tables S3-S12). According to a first phylogenetic analysis with all helminth genomes available in WormBase ParaSite [22], the STE11 family member D915_005277 may be specific for the order Plagiorchiida, while the CAMK family member D915_001682 may be specific for the family of Fasciolidae (Supplementary data 2/Fig. 11,12). Also the CMGC group kinase FhPK_0027 had no clear schistosome homolog, but appeared related to cyclin-dependent kinases (Smp_080730, D915_002458), based on full protein sequences and phylogenetic analysis (Supplementary data 2/Fig. 5). Finally, FhPK_0018 (PK group Others) had no schistosome homolog, but may represent an incomplete kinase sequence, which has likely complicated the homology search.

### *F. hepatica* kinases with homology to human kinases as basis for drug repurposing

On the one hand, identification of trematode-specific PKs and PKs with a particularly low sequence identity to the human orthologous gene could be desirable, given that therapeutic targeting should be possible without risking off-target effects in the human host. We identified the closest human homolog for *F. hepatica* PKs by global pairwise analysis and obtained percentages of identity and similarity for the protein kinase domains. In total, 23 PKs had a particularly low conservation of kinase domains between *F. hepatica* and humans (< 30% identity) (Table 2). The highest number of such genes was found in the PK group “Others”, such as SCY1 Like Pseudokinase 3 (SCYL3, 8.5% identity) and p53-related protein kinase (PRPK, 13.5% identity).

On the other hand, high sequence similarity between human and parasite PKs can facilitate a drug repurposing approach, in which available inhibitors of human PKs may show high potency against parasite PKs. Human homologs to parasite PKs with the highest PK domain identity (> 75%) are summarized in Table 3. A total of 16 PKs were found, which belong to the AGC (3 genes), CAMK (4 genes), CK1 (2 genes), CMGC (6 genes), and STE (1 gene) PK groups. The highest identity and similarity values (84.1% and 94%, respectively) were found for a microtubule affinity regulating kinases 1 homolog (MARK1, D915_002194), a member of the CAMK group.

**Table 3:** Summary of human homologs to *F. hepatica* PKs with PK domain sequence identity > 75%

PK group	*Fh*_Identifier	*Hs*_identifier	% Sequence identity	% Sequence similarity
CAMK	D915_002194	MARK1	84.1	94.0
CMGC	D915_004801	CK2a1	83.9	92.7
AGC	D915_000165	PKACa	81.6	91.8
STE	D915_001478	PAK3	80.6	90.9
CK1	FhPK_0002	CK1g3	80.3	89.0
CAMK	D915_003411	MARK2	78.2	90.5
CMGC	D915_003190	GSK3B	77.9	90.2
CAMK	D915_001485	SNRK	77.6	86.2
AGC	D915_004859	RSK2	77.5	87.0
CK1	FhPK_0011	CK1d	76.8	85.9
CMGC	D915_002401	PITSLRE	76.1	87.7
CMGC	D915_007819	DYRK2	75.9	84.8
CAMK	D915_001295	BRSK2	75.8	85.3
CMGC	D915_006769	CDK5	75.4	89.1
CMGC	D915_000427	CK2a1	75.2	89.9
AGC	D915_008611	PKCi	75.0	85.8

**Fig. 4:**
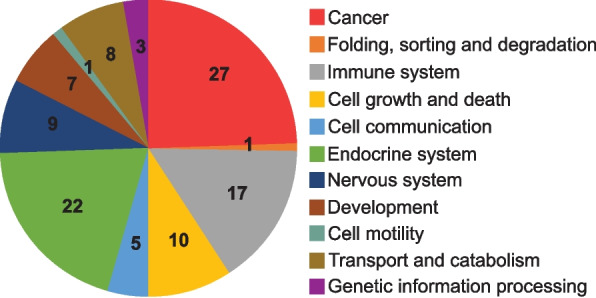
KEGG-associated pathways of *F. hepatica* PKs annotated in the KEGG databases. Numbers represent the number of pathways. Associated genes are listed in Supplementary data 3

The 245 *F. hepatica* PKs were subjected to KEGG functional annotation via BlastKOALA based on human orthology. A total of 110 PKs (44.9% of all PKs) were annotated by KEGG and were allocated to eleven associated pathways (Fig. 4 and Supplementary data 3). Based on the KEGG terms, most PK functions were associated with Cancer (24.54%), Endocrine system (20%), Immune system (17.45%), and Cell growth and death (9.09%). Taken together, these data open the path to make use of inhibitors designed to target human PKs, e.g. developed for anti-cancer therapy, in a drug-repurposing approach against *F. hepatica*.

### *In-vitro* screening of PK inhibitors against *F. hepatica*

To test if a drug repurposing approach could successfully deliver PK inhibitors with anti-*Fasciola* activity, we focused on inhibitors that are approved for human use or part of clinical trials (Supplementary data 2/Table 6) to cure human diseases, and for which orthologs to the human target were present in our *F. hepatica* kinome dataset. We selected the following small-molecule PK inhibitors: vandetanib (ZD6474) [56], foretinib (XL880), tyrosine kinase-IN-1 (XL999) and ruboxistaurin (LY333531). Ruboxistaurin is an isoform-specific inhibitor of PKCβ [57]. Within the *F. hepatica* kinome, one protein was annotated as PKCβ (D915_006901) and we previously demonstrated in vitro activity of ruboxistaurin against the adult stage of *F. hepatica*, but had not tested its full potency against younger stages [58]. The other compounds are multi-kinase inhibitors targeting several RTKs. Vandetanib inhibits human endothelial growth factor receptors (VEGFR2/3), RET, EGFR, and FGFR [59] and is in use as anti-cancer drug [60]. Foretinib inhibits RTKs involved in cancer development (VEGFR2, PDGFR‐β, MET, RON, ERK and FLT3) [61], while TK-IN-1 is known to inhibit human FGFR 1/3, PDGFRα/β, KIT, VEGFR2, and FLT3 [62]. While we did not find VEGFR or PDGFR orthologs in the *F. hepatica* kinome, we identified three proteins (FhPK_0030, D915_000912, and D915_003452) belonging to the EGFR subfamily, and four proteins (D915_003390, D915_003619, D915_005046, and D915_007307) belonging to the FGFR subfamily. The multi-RTK inhibitors were selected due to their broad-spectrum activity and potential to disrupt key signalling pathways that are conserved across species, making them promising candidates for antiparasitic in vitro investigations. All putative targets in *F. hepatica* were associated with the cancer pathway according to KEGG functional analysis (Supplementary data 3).

**Fig. 5:**
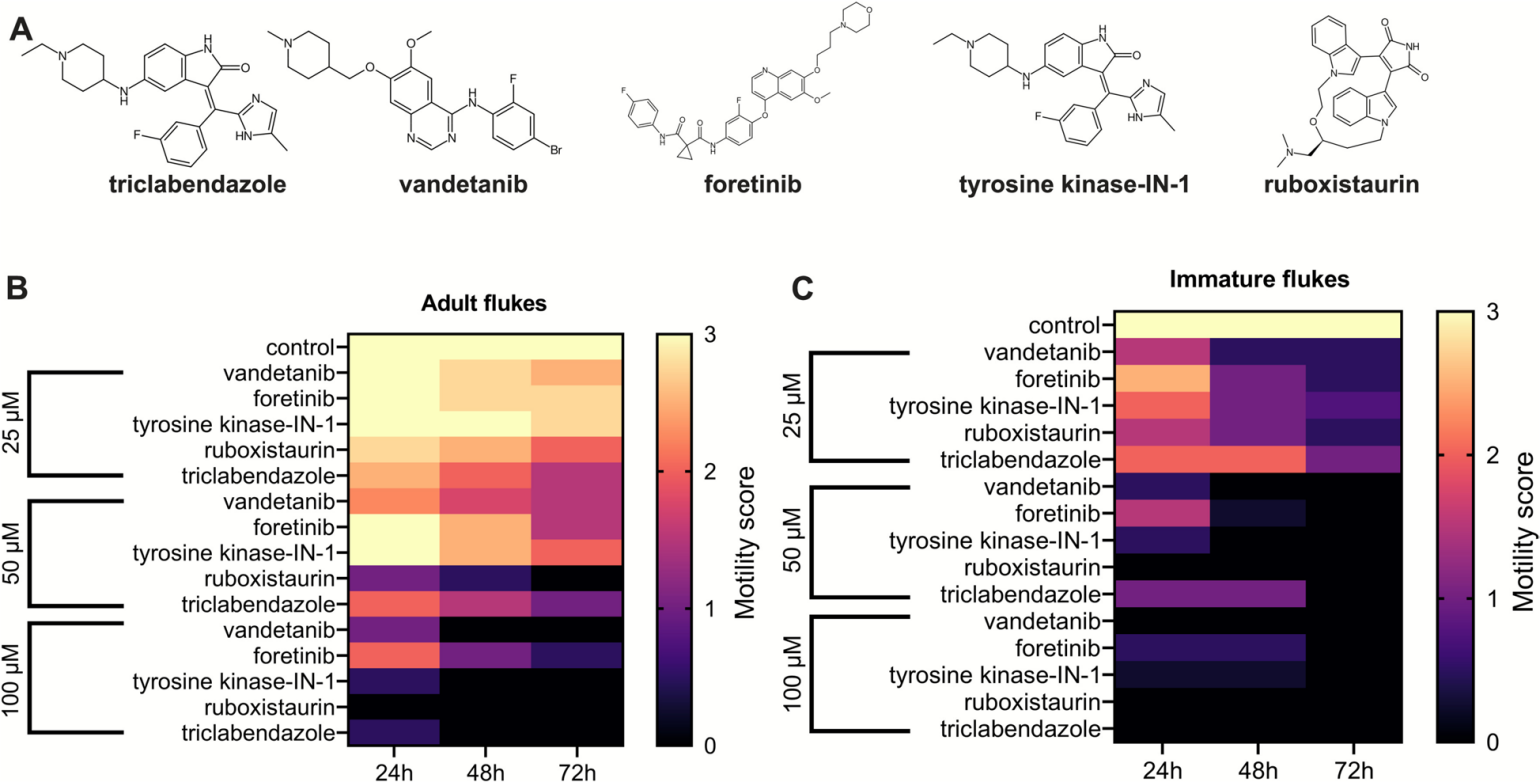
Heatmaps illustrating the motility scores of *F. hepatica* following exposure to four protein kinase (PK) inhibitors compared to triclabendazole. **A** Structures of tested compounds. **B** Motility scores for adult flukes and **C** motility scores for immature flukes are shown. The flukes were cultured for 72 h, with fresh medium containing the respective PK inhibitors or DMSO as negative control. Motility was assessed at regular intervals of 24 h, and scores were assigned based on the following criteria: 0 = dead, 1 = minimal movement, 2 = reduced movement, and 3 = normal movement. Statistical data are summarized in Supplementary data 2

The inhibitors were tested at concentrations of 25 µM, 50 µM, and 100 µM in vitro against *F. hepatica*. We targeted both stages relevant for pathology in the mammalian host: immature worms causing acute disease, and adult worms that are involved in chronic disease. Fluke survival and motility were assessed every 24 h over a 72 h culture period (Fig. 5 and Supplementary data 2). As a reference, we treated worms with TCBZ under the same conditions. For all inhibitors and concentrations, immature flukes were more sensitive than adults, i.e. their motility was more strongly reduced (score between 0.5 and 1.0) compared to the control group (score 3). After 24 h treatment with the lowest test concentration of 25 µM, vandetanib was most potent against immature flukes followed by ruboxistaurin. Both inhibitors lowered motility of worms by about 50% compared to control worms. After extended exposure of 72 h, both inhibitors as well as foretinib reduced worm motility to a minimum level, while TK-IN-1 was slightly less potent. At 50 µM, ruboxistaurin was the most effective inhibitor and killed all worms within the first 24 h, while the three other inhibitors were lethal after 48–72 h of treatment. The reference drug TCBZ needed higher concentrations and/or longer treatment periods to achieve the same effect.

In case of the adult parasite stage, all PK inhibitors as well as TCBZ only mildly reduced motility at 25 µM (score between 1.5 and 2.5). At 50 µM, ruboxistaurin was able to kill adult worms within 72 h, and after 24 h, motility was already severely affected. TCBZ was sublethal (score 1) at the same concentration and only slightly (score 1.5) more potent in reducing motility than vandetanib. The two other PK inhibitors were less potent and caused a motility decrease either late (foretinib) or overall only weakly (score 2) (TK-IN-1). At 100 µM, all worms were killed by ruboxistaurin within the first 24 h, and by vandetanib, TK-IN-1 and TCBZ within 48 h of culture. TK-IN-1 and TCBZ caused parasite death in some individuals within 24 h of exposure. In contrast, worms survived foretinib treatment even for 72 h.

Even at a low concentration, all the selected small-molecule PK inhibitors were more effective than the reference drug TCBZ on immature flukes. Contrary to this, against adults, ruboxistaurin was the only PK inhibitor that caused a more pronounced reduction in motility of the worms compared with TCBZ. Taken together, the PKCβ inhibitor ruboxistaurin stood out with its potency in killing both parasite stages within a short treatment period. Vandetanib also demonstrated a strong and fast activity, especially against immature worms.

## Discussion

### Achieving kinome curation of a neglected pathogen

The aim of this study was to obtain an overview of PKs present in the liver fluke *F. hepatica* in order to provide a solid basis for future investigations of drug targets and pathogen cell biology. We identified a kinome dataset comprising 245 PKs followed by a drug repurposing approach to find commercially available small-molecule inhibitors with activity against this parasite.

In the past, to curate the kinomes of other parasitic species, numerous studies relied on identifying kinase sequences through the construction of Hidden Markov Models (HMMs) [63] utilising databases like Pfam [31] or Position Specific Scoring Matrix (PSSM) [64] from the Conserved Domain Database (CDD). However, the combination of these methods, rather than individual use, can significantly enhance the prediction and classification of PKs. The program Kinannote employs a combination of HMMs and PSSM to identify and classify PKs with a specificity and precision exceeding 70% and 80%, respectively [23]. Nevertheless, there are some limitations associated with this program, leading to incomplete classification of some PKs [65]. The integration of independent phylogenetic analysis can help surmount limitations in kinome curation. In this study, we employed such a modified bioinformatic pipeline that predicted, curated, and classified the kinome of *F. hepatica*. This procedure improved the overall PK number from the Kinannote-based draft kinome of 208 ePKs to the final kinome of 241 ePKs and four aPKs.

The rather low BUSCO score (71%) combined with 9.5% fragmented genes in the *F. hepatica* genome annotation posed a challenge in fully annotating the PKs. While our kinome comparison to *S. mansoni* and *S. haematobium* revealed four schistosome-unique PKs for which no *Fasciola* homolog was found, it needs to be considered that some of these genes might have been missed due to gene fragmentation in the *F. hepatica* genome. More PKs may be discovered with improved genome quality, e.g. by long-read sequencing.

### Prominent kinome groups and families in *F. hepatica* and comparison to *S. mansoni*

Among the nine ePK groups defined for the *F. hepatica* kinome, we like to highlight the CMGC and the TK groups, in which several members have been previously found to be important for the development of related blood flukes, but were also discussed as interesting antiparasitic targets. The largest PK group identified in *F. hepatica* was CMGC with 43 members (17.84% of total ePKs) including seven MAP kinases. The MAPK signaling pathway is essential in regulating cellular processes such as cell proliferation, cell differentiation and apoptosis [66]. Within the kinome of *F. hepatica*, we found all major members of the MAPK cascade. In *S. mansoni*, RNAi-mediated functional analysis of the MAPK family members SmJNK and SmERK1 revealed a developmental role in adult worms [12, 67]. The kinase domain of FhJNK (D915_006573) exhibits 69.7% identity and 81.5% similarity to SmJNK (Smp_172240), and FhERK1 homologs (D915_001614 and D915_006871) exhibit even 83.1% and 90.7% identity and 83% and 92.4% similarity, respectively, to the SmERK1 homologs (Smp_047900 and Smp_142050) (Supplementary data 1/Table S7). Recent work from the Falcone lab demonstrated that small-molecule inhibitors of schistosome JNK and ERK displaying anti-schistosomal activity can be successfully derived from docking-based virtual screening [68, 69]. This could be a valuable approach also in the search for new anti-*Fasciola* compounds and the high sequence similarity may enable finding candidates with broad-spectrum activity against both parasite groups.

The TK group comprised 17 receptor tyrosine kinases (RTK) and 16 cytoplasmic tyrosine kinases (CTK) in *F. hepatica*. Receptor tyrosine kinases (RTKs) are a group of membrane-bound receptors that respond to external cues such as growth factors [50, 51]. RTKs function as signal transducers, mediating cell-to-cell communication. RTKs phosphorylate tyrosine residues on key intracellular substrate proteins and thereby influence cell fate, including proliferation, migration and differentiation [70]. Amongst others, RTKs take influence on members of the MAPK signalling cascade mentioned above [71, 72]. A RTK family unique for invertebrates includes venus kinase receptors (VKRs), which have a tyrosine kinase domain resembling the one of insulin receptors [73]. In *S. mansoni*, VKRs have a dominant role in reproduction, egg development, and larval development [74–76]. Gene silencing revealed a contribution of SmVKR1 and SmVKR2 to gametogenesis [12, 76] and in addition, SmVKR1 (Smp_019790) was found to interact with a multikinase complex consisting of the Src-kinase SmTK3 and the Src/Abl hybrid-kinase SmTK6, which regulates cell growth, mitosis, and cytoskeleton functions [77]. The phylogenetic analysis between *S. mansoni* and *F. hepatica* identified FhPK_0008 and D915_003706 as homologs to SmVKR1 and SmVKR2, respectively, which exhibit 78.2–78.4% identity and 86.6–88% similarity to the schistosomal kinase domains. While kinase research in *F. hepatica* is just in its infancy compared to schistosomes, it will be highly interesting to compare functional aspects of PK signalling between both evolutionary related, yet different parasites. One obvious difference is their reproduction, given that liver flukes are hermaphrodites, whereas blood flukes reproduce as dioecious parasites.

### Strategies to derive anti-parasitic targets and inhibitors from the *F. hepatica* kinome

With a curated kinome of *F. hepatica* at hand, it is possible to prioritize potential therapeutic targets. Our analysis revealed homologous PKs that were shared between the trematode kinomes but lack homologs in the human kinome. In addition, several PKs with a low degree of sequence conservation between *F. hepatica* and humans were obtained. Both types could be attractive for designing drugs with reduced side effects, because such drugs are less likely to provoke off-target effects in the human host. For instance, invertebrate-specific VKRs play crucial roles in lifecycle progression and their absence in humans makes them potential drug targets against fascioliasis. Future work should focus on the functional characterization of such parasite-unique PKs in order to identify genes that are important for liver fluke survival. Recombinant expression of these PKs could then open the way for high-throughput screenings of compound libraries to identify candidate inhibitors.

Furthermore, our analysis revealed PKs that are highly conserved between parasites and humans. Repurposing of already available kinase inhibitors, which are in use for the treatment of human diseases, is another strategy previously discussed in anthelminthics research [19, 78, 79]. To narrow down the list of candidate inhibitors, this can be combined with in silico prediction of the essentiality of the orthologous targets of such inhibitors in the pathogen. All four candidate inhibitors evaluated in our study against *F. hepatica* were predicted to be active against the related parasite *S. haematobium* based on a previous in silico interrogation of its kinome. For these inhibitors, essentiality of their targets for parasite survival was suggested by lethal knockout phenotypes in model organisms and mapping to unique chokepoints in key biological pathways [11]. Although antiparasitic activity of the predicted compounds was not experimentally assessed against *S. haematobium*, this would be an interesting future task, since we found here in vitro activity for some of the compounds against *F. hepatica*.

A KEGG-based functional annotation of the *F. hepatica* PKs revealed that out of 110 KEGG-annotated PKs, 24.54% were associated to human orthologs that are part of cancer pathways. Many PK inhibitors exhibiting anti-cancer properties are known to target the proliferation of cancer stem cells [80, 81]. Stem cells may also represent a key to control parasitic worms [82] and thus, it may be fruitful to evaluate relevant PK inhibitors in vitro for their capacity to hinder growth, development and/or viability of liver flukes. Eventually, repurposing of approved small-molecule PK inhibitors could significantly reduce the time needed towards a new drug for the treatment of fascioliasis. Certainly, targeting a pathogen protein and host protein at the same time bears the risk of unwanted side-effects in the host. By selecting inhibitors that successfully completed clinical studies and were proven to be safe, as in our pilot screen of inhibitors, this risk can be minimized.

### Repurposed PK inhibitors against *F. hepatica*

To test the hypothesis that repurposing of existing small-molecule inhibitors represents a successful strategy in finding new fasciolicidal compounds, we selected four candidate inhibitors. We had prioritized vandetanib, foretinib and TK-IN-1 as anti-cancer drugs that target multiple RTKs. Poly-pharmacology is a strategy in which a single molecule targets more than one kinase and may have benefits by increasing efficacy and mitigating the development of resistance to the kinase inhibitor [83]. Cancer is often associated with deregulation of RTKs like EGFR, FGFR, or VEGFR and accordingly, several RTK-targeting inhibitors have been developed for biomedical use [50]. Ruboxistaurin, our fourth candidate, is a selective inhibitor of PKCβ1 (IC_50_ = 4.7 nM) and PKCβ2 (IC_50_ = 5.9 nM) [57], but at higher concentrations has also inhibitory effects on PDK1, which is involved in the insulin-like growth factor signalling pathway [84]. Our in vitro tests revealed strong effects for vandetanib and ruboxistaurin against both immature and adult *F. hepatica*, which were comparable or even higher to TCBZ at the same test concentration in vitro.

Ruboxistaurin has passed phase III clinical trials for diabetic retinopathy [85–87]. PKC isoforms are key players downstream of TCR and BCR signalling [88] and in addition, mediate negative regulation of MAPK signalling [89]. Furthermore, human PKCs are involved in the regulation of muscle contraction, cell migration and focal adhesion [90]. Indeed, when isolated muscle strips of *F. hepatica* were exposed to the PKC activator phorbol 12-myristate 13-acetate (PMA), contraction frequency and amplitude increased [91]. We annotated one protein as PKCβ (D915_006901). In our recent spatial transcriptomics-based analysis of *F. hepatica* adult worms, we found PKCβ predominantly expressed close to the surface of the worm (tegument or sub-tegumental musculature) and a reduction of vitality when adult worms were treated with ruboxistaurin [58]. We now extended this finding and could demonstrate potent activity also against the immature parasite stage. With a tolerated dose of 800 mg/kg body weight in rats [92], future studies may test the potency of ruboxistaurin in reducing worm burden in vivo. While a final proof of PKCβ as target of ruboxistaurin in *F. hepatica* may be obtained by enzyme activity assays, the high percentage of similarity of human and parasite PK domains (86.8%) makes an interaction very likely.

Almost as potent as ruboxistaurin was the multi-RTK inhibitor vandetanib. That vandetanib was more potent than the two other multi-RTK inhibitors may have various reasons, such as a higher importance of the particular kinases targeted by vandetanib for fluke survival, or different uptake kinetics of the compound into the parasites. Vandetanib is an inhibitor of human VEGFR-2 (KDR) [60], but also inhibits the growth factor receptors EGFR and FGFR [59]. Vandetanib significantly reduced the vitality of *F. hepatica* in our in vitro tests. The absence of VEGFR receptors in invertebrates [93] suggests that metazoan VEGFR, PDGFR and FGFR had a common RTK ancestor during evolution [94]. A gene duplication event gave rise to FGFR and VEGFR/PDGFR-like genes. Thus, with FGFR as the only representative in invertebrates including *F. hepatica*, we speculate that FGFRs or EGFR family members are the targets of vandetanib. Vandetanib is an FDA-approved drug for treatment of thyroid cancer and has an excellent oral bioavailability [95–97]. Importantly, vandetanib was able to reduce the worm burden in a mouse model of *S. mansoni* infection by 48% with a single oral dose of 400 mg/kg body weight [98]. This together with the high fasciolicidal activity in our in vitro assay encourages the assessment of vandetanib as another prime candidate for in vivo drug studies against liver flukes. Since vandetanib is a multi-RTK inhibitor, it would be interesting to figure out which of the targeted PKs are vital for the parasite and to assess the cellular downstream consequences of inhibition in *F. hepatica*. This knowledge may spotlight even more efficient ways to kill the parasite.

The clinical use of PK inhibitors in ruminants and in veterinary medicine in general is still rare. This may have various reasons, including the unavailability of generic versions of available human inhibitors, in case of drug repurposing strategies, which makes treatments expensive [99]. Nevertheless, some studies repurposed human tyrosine kinase inhibitors to treat cancer in dogs [100], and a kinase inhibitor used against an apicomplexan parasite was save even in pregnant sheep and calves, with absence of toxicity after administration [101, 102]. This suggests that repurposing PK inhibitors for animal medicine or de novo kinase inhibitor discovery may represent a fruitful strategy in veterinary medicine, including treatment of fasciolosis.

## Conclusion

In this study, we curated a kinome dataset of *F. hepatica*, which could be utilized as a basis to study the role of kinases for the biology of *F. hepatica*, such as their essentiality for parasite development or reproduction. Furthermore, with the availability of this kinome, the prioritization and discovery of potential druggable kinases will become easier. The obtained percentages of identity and similarity between kinase domains of three trematode species and the human host suggest that there is a potential for both strategies: trematode-specific drug development and drug repurposing of PK inhibitors from human biomedical research. Successful in vitro tests with the PKCβ inhibitor ruboxistaurin (cleared phase III clinical trials) and the multi-RTK inhibitor vandetanib (FDA-approved) against *F. hepatica* underline the potential of a drug repurposing strategy. We see the kinome as a suitable starting point for the search of new potential druggable targets in order to overcome parasitic infection.

## Supplementary Information

Additional file 1: Supplementary data 1. XLSX file containing supplementary tables 1–12 presenting the *Fasciola hepatica* kinome. Table S1: The *F. hepatica* kinome, kinome classification, amino acid sequences as well as functional annotations with InterPro, Pfam and SUPERFAMILY domain ID and their description, KEGG terms as well as their definitions. Table S2: Curated *F. hepatica* protein sequences with assignment of PK families based on phylogenetic analysis. Tables S3−12: Closest homolog of *F. hepatica* in *S. mansoni*, *S. haematobium* and *H. sapiens* kinomes based on global pairwise analysis results via EMBOSS needle for the PK domains in the PK groups AGC (S3), Atypical PKs (S4), CAMK (S5), CK1 (S6), CMGC (S7), Others (S8), RGC (S9), STE (S10), TK (S11), TKL (12). Identity and similarity (in %) of similar regions in the protein kinase domain sequences of genes with highest bit-scores compared to the *F. hepatica* gene are indicated, with an exception of using full length sequences instead of PK domains for Atypical PKs. Note that the genes are not necessarily orthologous genes. In case no homolog was identified (NA), results of an additional BLAST search with full-length kinase sequences are presented.

Additional file 2: Supplementary data 2. Supplementary Figs. 1–10 depict a phylogenetic tree of each individual PK group including the posterior probabilities: AGC (Supplementary Fig. 1), Atypical (Supplementary Fig. 2), CAMK (Supplementary Fig. 3), CK1 (Supplementary Fig. 4), CMGC (Supplementary Fig. 5), Others (Supplementary Fig. 6), RGC (Supplementary Fig. 7), STE (Supplementary Fig. 8), TK (Supplementary Fig. 9), and TKL (Supplementary Fig. 10). Supplementary Fig. 11 depicts a phylogenetic tree for the STE11-family member D915_005277 and Supplementary Fig. 12 depicts a phylogenetic tree for the CAMK-family member D915_001682. Supplementary Tables 1–5: Statistical data on motility scores of the treated adult and immature flukes depicted in Fig. 5. Suppl. Table 1: The statistical values for control *vs.* vandetanib treated adult and immature flukes. Table 2: The statistical values for control *vs.* foretinib treated adult and immature flukes. Table 3: The statistical values for control *vs.* tyrosine kinase-IN-1 treated adult and immature flukes. Table 4: The statistical values for control *vs.* ruboxistaurin treated adult and immature flukes. Table 5: The statistical values for control *vs.* triclabendazole treated adult and immature flukes. Table 6: The clinical trial status of protein kinase inhibitor used in vitro against immature and adult flukes.

Additional file 3: Supplementary data 3. KEGG pathway reconstruction for the kinases in the *F. hepatica* kinome.

Additional file 4: Supplementary data 4. Protein kinase homologs in *F. hepatica* and *S. mansoni* predicted by the phylogenetic tree in Fig. 2.

Additional file 5: Supplementary video 1. Adult *F. hepatica* with normal motility (score 3).

Additional file 6: Supplementary video 2. Adult *F. hepatica* with moderately reduced motility (score 2).

Additional file 7: Supplementary video 3. Adult *F. hepatica* with severely reduced motility (score 1).

Additional file 8: Supplementary video 4. Adult *F. hepatica* with absent motility (score 0, dead).

Additional file 9: Supplementary video 5. Immature *F. hepatica* with normal motility (score 3).

Additional file 10: Supplementary video 6. Immature *F. hepatica* with moderately reduced motility (score 2, fluke at bottom; score 3, fluke at top).

Additional file 11: Supplementary video 7. Immature *F. hepatica* with severely reduced motility (score 1); sporadic movements at the anterior end.

Additional file 12: Supplementary video 8. Immature *F. hepatica* with absent motility (score 0, dead).

## Data Availability

All data generated or analysed during this study are included in this published article and its supplementary information files.
