# Supplementary material for: Curation of the Fasciola hepatica kinome as a resource for drug target discovery
Source: BMC Genomics. 2026 Jan 16;27:98. doi: 10.1186/s12864-025-12513-w (PMC12837073; doi:10.1186/s12864-025-12513-w)
Supplement: Supplementary file 2 — Additional file 2: Supplementary data 2. Supplementary Figs. 1–10 depict a phylogenetic tree of each individual PK group including the posterior probabilities: AGC (Supplementary Fig. 1), Atypical (Supplementary Fig. 2), CAMK (Supplementary Fig. 3), CK1 (Supplementary Fig. 4), CMGC (Supplementary Fig. 5), Others (Supplementary Fig. 6), RGC (Supplementary Fig. 7), STE (Supplementary Fig. 8), TK (Supplementary Fig. 9), and TKL (Supplementary Fig. 10). Supplementary Fig. 11 depicts a phylogenetic tree for the STE11-family member D915_005277 and Supplementary Fig. 12 depicts a phylogenetic tree for the CAMK-family member D915_001682. Supplementary Tables 1–5: Statistical data on motility scores of the treated adult and immature flukes depicted in Fig. 5. Suppl. Table 1: The statistical values for control vs. vandetanib treated adult and immature flukes. Table 2: The statistical values for control vs. foretinib treated adult and immature flukes. Table 3: The statistical values for control vs. tyrosine kinase-IN-1 treated adult and immature flukes. Table 4: The statistical values for control vs. ruboxistaurin treated adult and immature flukes. Table 5: The statistical values for control vs. triclabendazole treated adult and immature flukes. Table 6: The clinical trial status of protein kinase inhibitor used in vitro against immature and adult flukes. [file 12864_2025_12513_MOESM2_ESM.pdf]

Supplementary data 2

Curation of the *Fasciola hepatica* kinome as a resource for drug target discovery

Sagar Ajmera[1], Oliver Puckelwaldt[1], Andreas J. Stroehlein[2#], Simone Haeberlein[1#*]

[1]Institute of Parasitology, BFS, Justus Liebig University, Giessen, Germany

[2]Department for Biological Safety, German Federal Institute for Risk Assessment, Berlin, Germany

[#]Shared senior authorship

*Contact, Correspondence: simone.haeberlein@vetmed.uni-giessen.de

Supplementary Figures 1 to 10. Phylogenetic analysis of protein kinase (PK) groups of *F. hepatica* and *S. mansoni*. Following the alignment of amino acid sequences representing AGC, Atypical, CAMK, CK1, CMGC, Others, RGC, STE, TK, and TKL groups, phylogenetic trees were constructed. High-resolution figures of individual trees including nodal support values (posterior probabilities) and sequence identifiers are given.

Supplementary Figure 11. Phylogenetic tree for the STE11family member D915_005277, possibly a Plagiorchiida-specific kinase. Tree was generated from all helminth genomes available in WormBase ParaSite (https://parasite.wormbase.org/index.html)

Supplementary Figure 12. Phylogenetic tree for the CAMK-family member D915_001682, possibly a Fasciolidae-specific kinase. Tree was generated from all helminth genomes available in WormBase ParaSite (https://parasite.wormbase.org/index.html)

Supplementary Tables 1 to 5. Statistical data on motility scores of the treated adult and immature flukes depicted in Figure 5.

Supplementary Table 6. The clinical trial status of protein kinase inhibitors used in vitro against immature and adult flukes.

Supplementary Figure 1. AGC kinase group

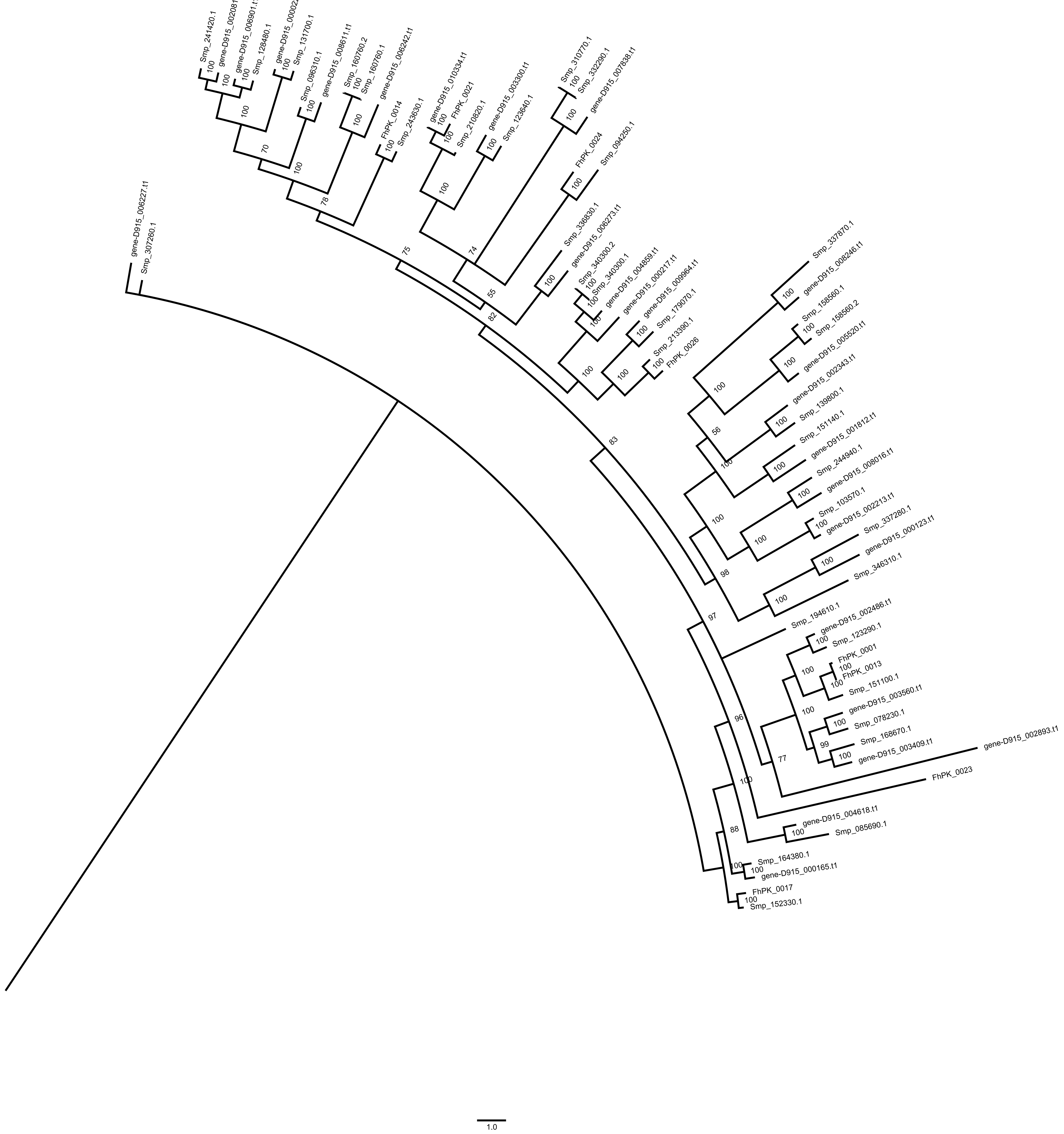

Supplementary Figure 2. Atypical kinase group

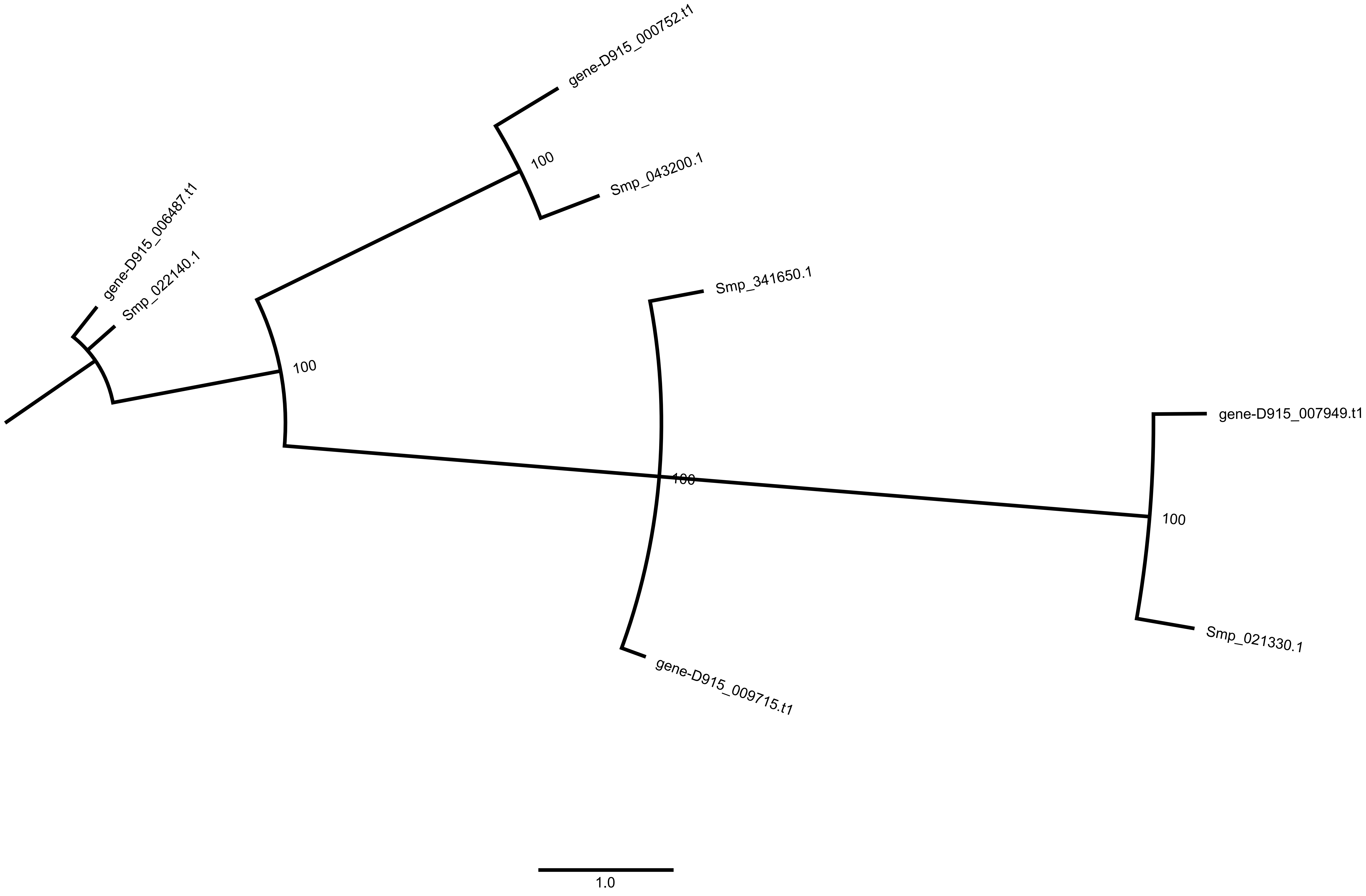

Supplementary Figure 3. CAMK kinase group

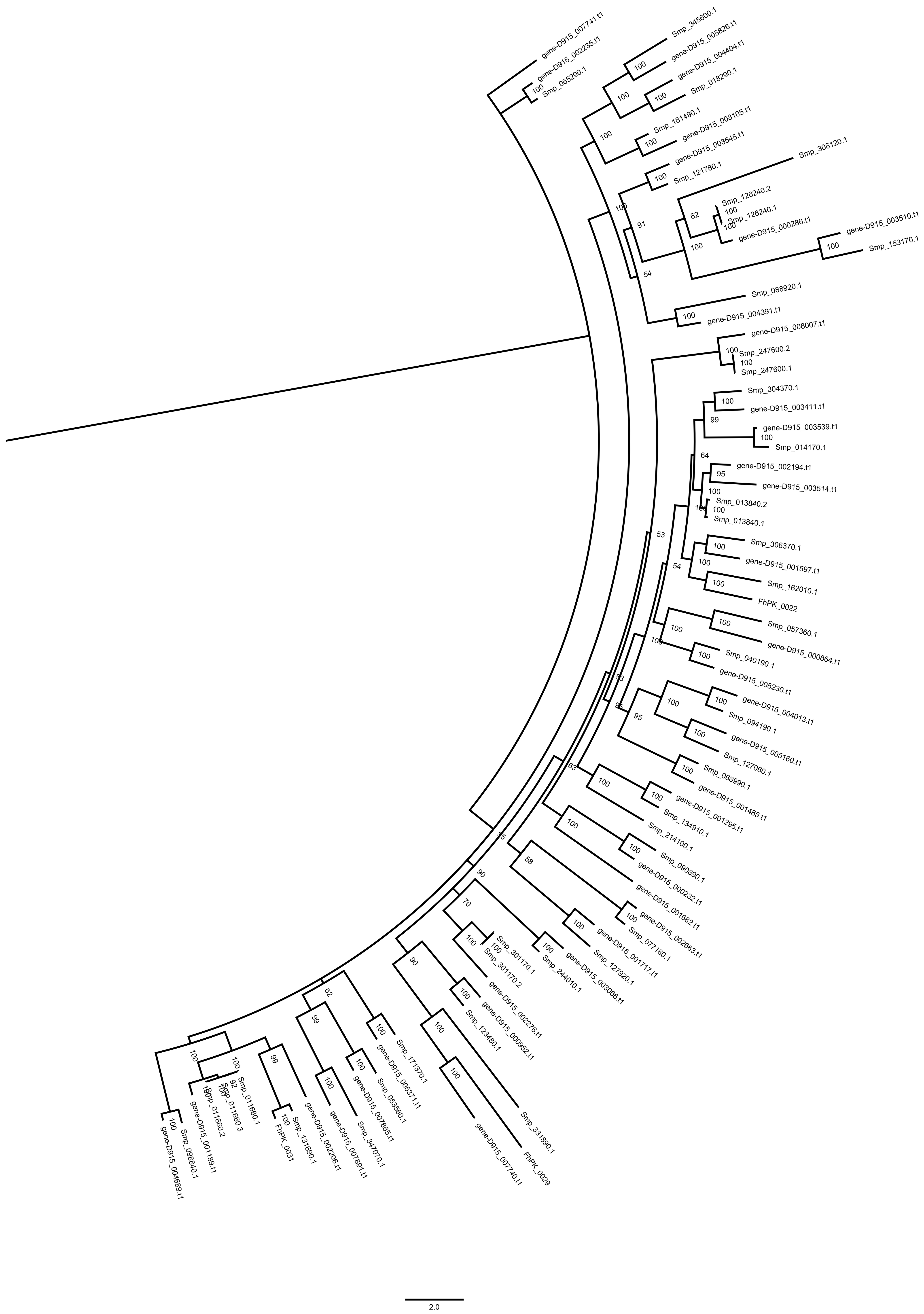

Supplementary Figure 4. CK1 kinase group

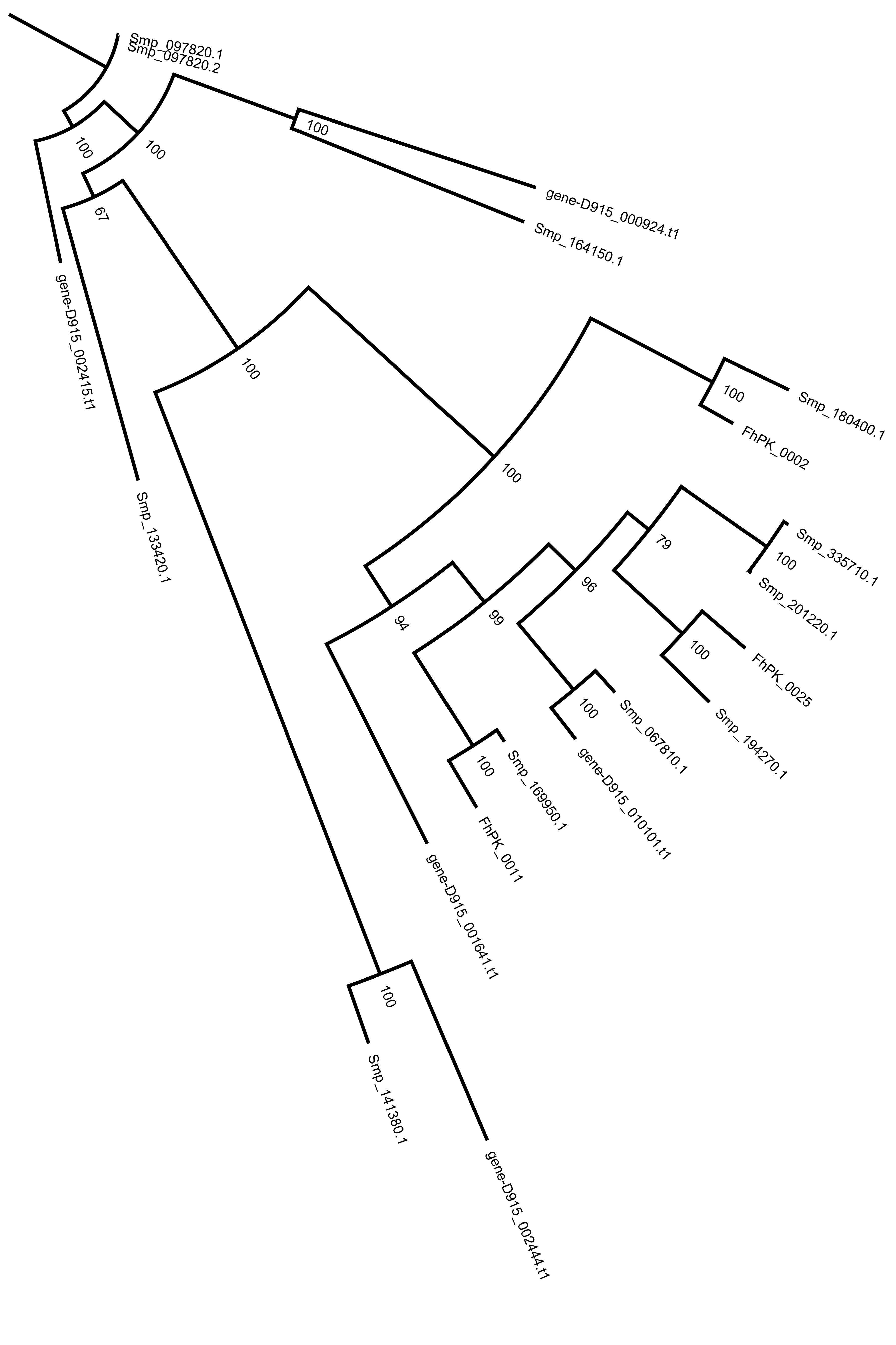

Supplementary Figure 5. CMGC kinase group

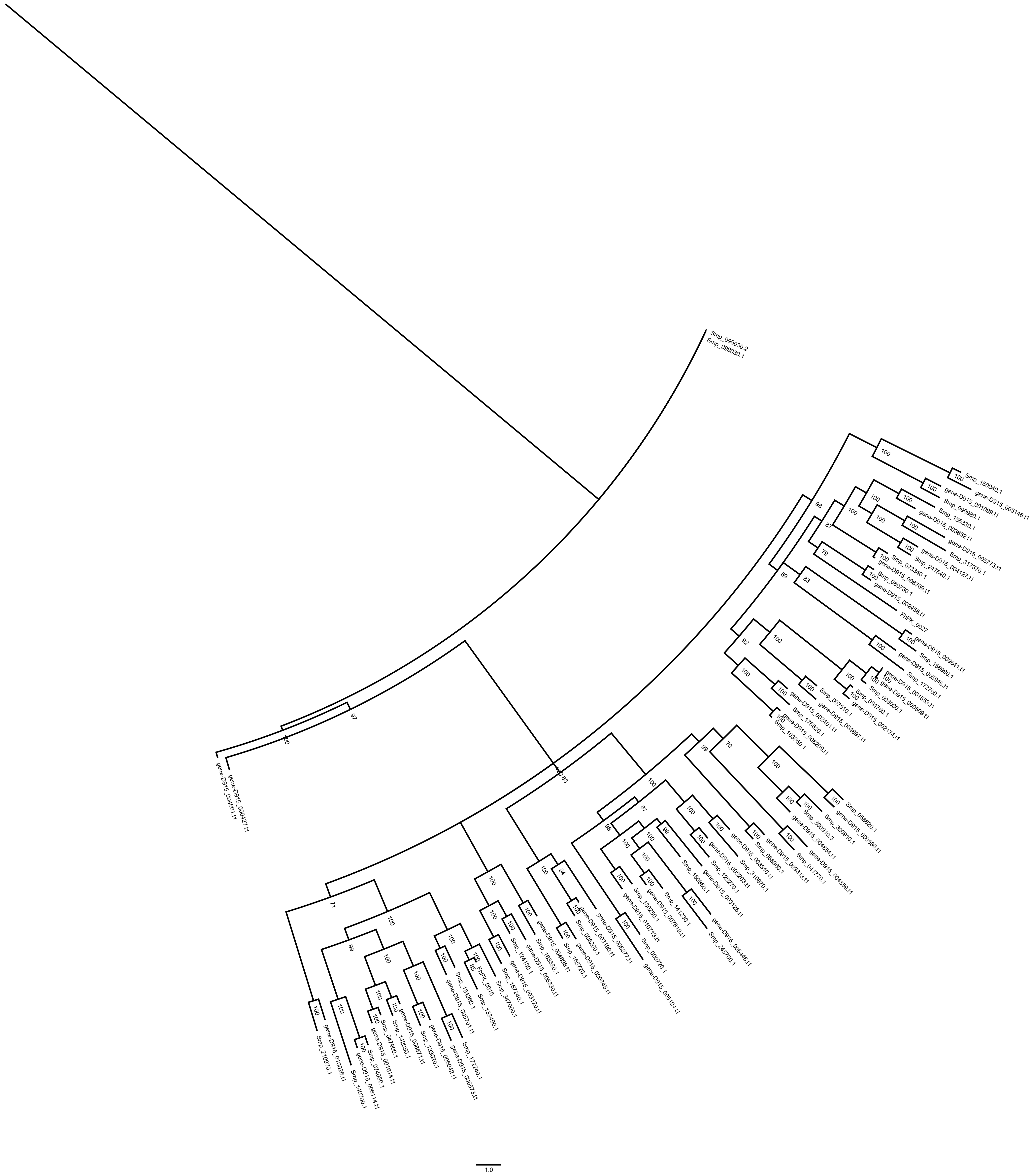

Supplementary Figure 6. Others kinase group

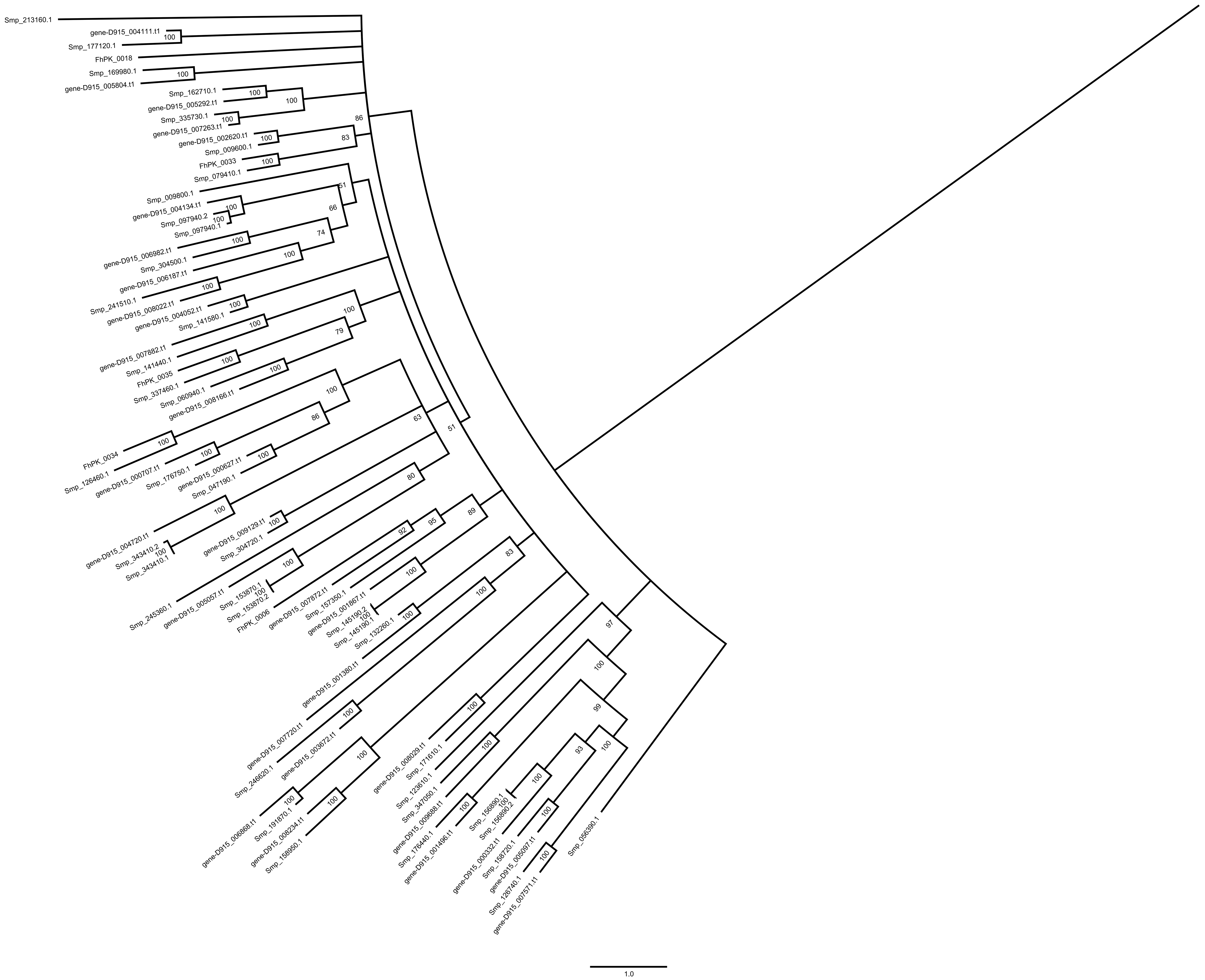

Supplementary Figure 7. RGC kinase group

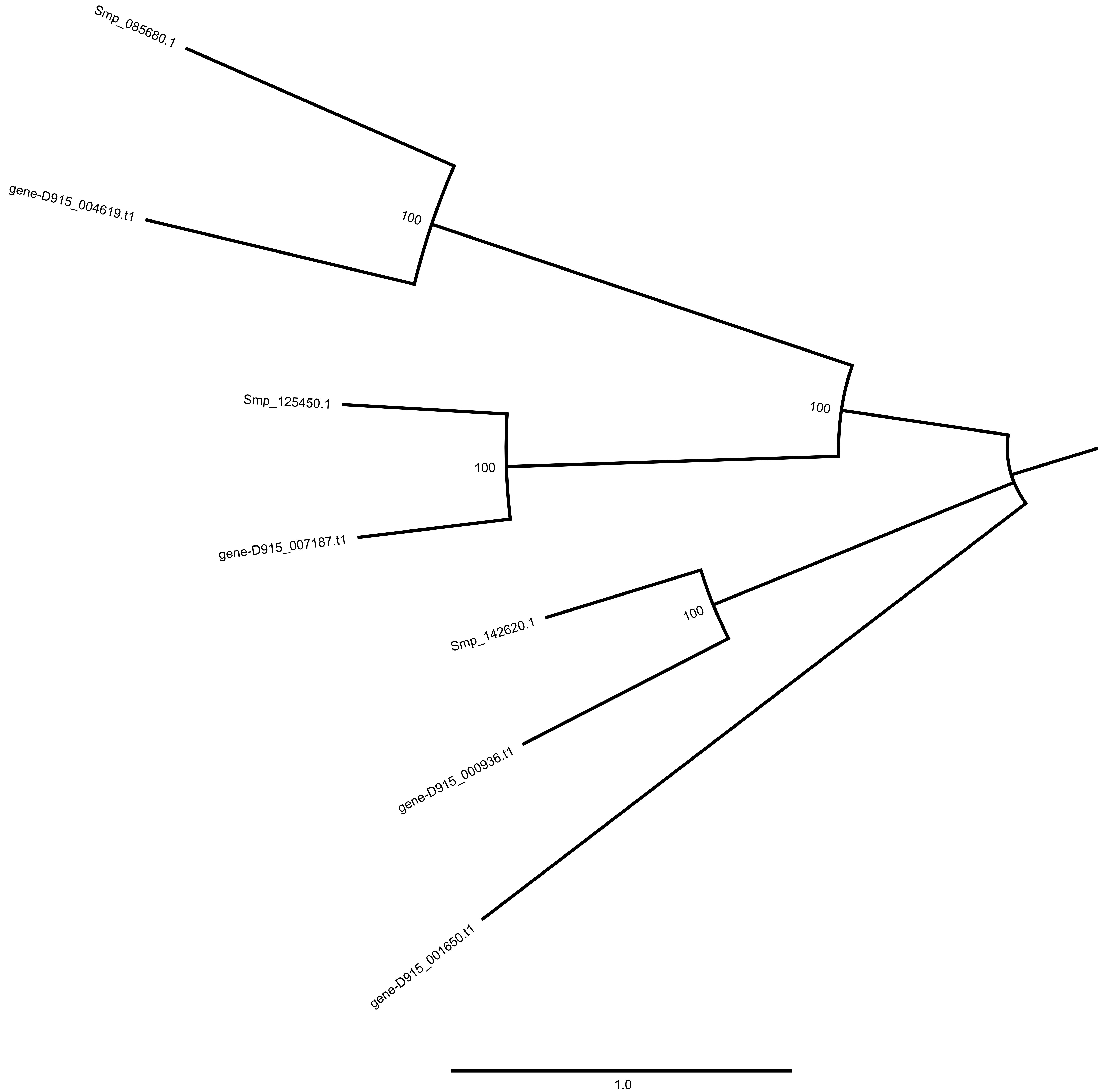

Supplementary Figure 8. STE kinase group

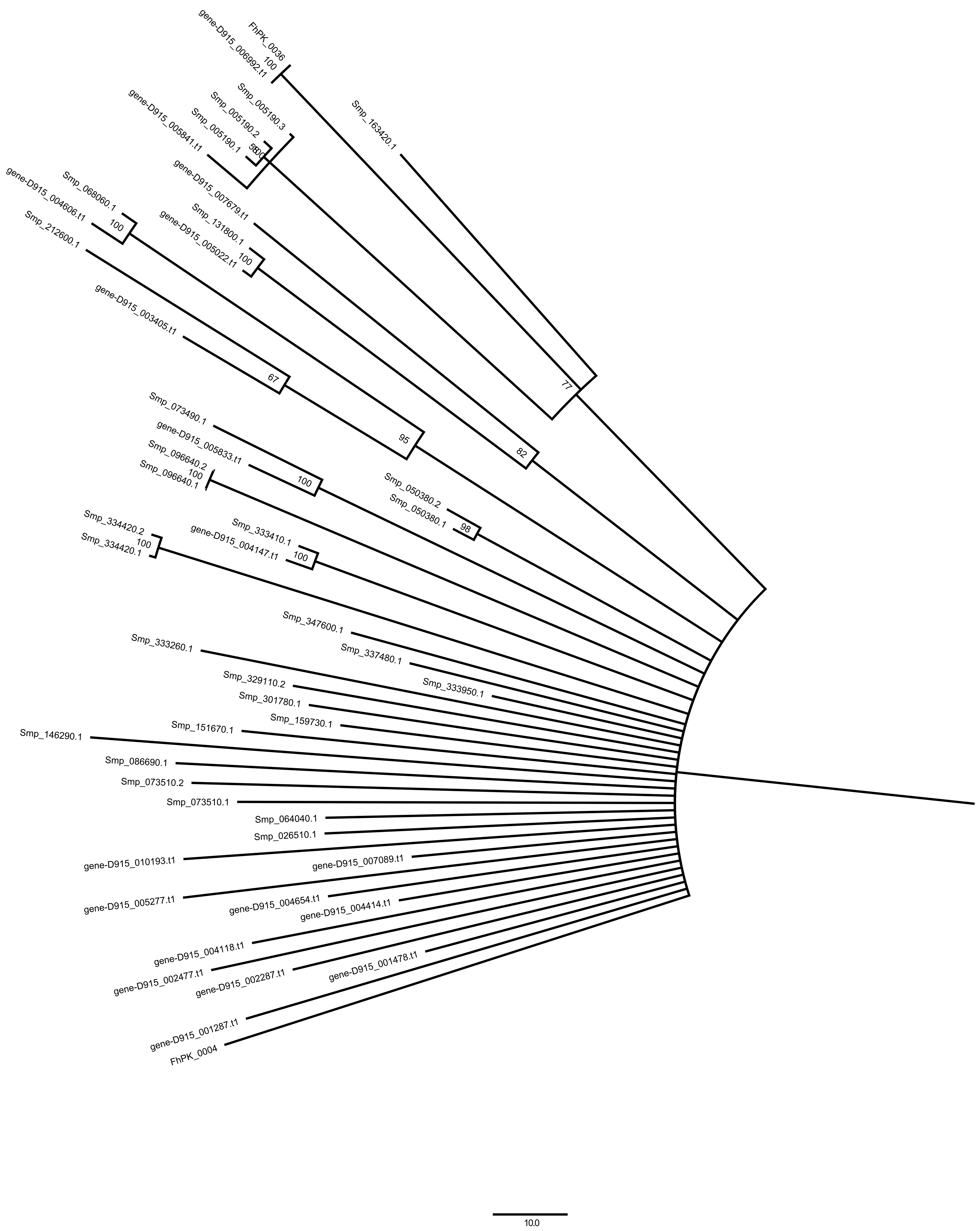

Supplementary Figure 9. TK kinase group

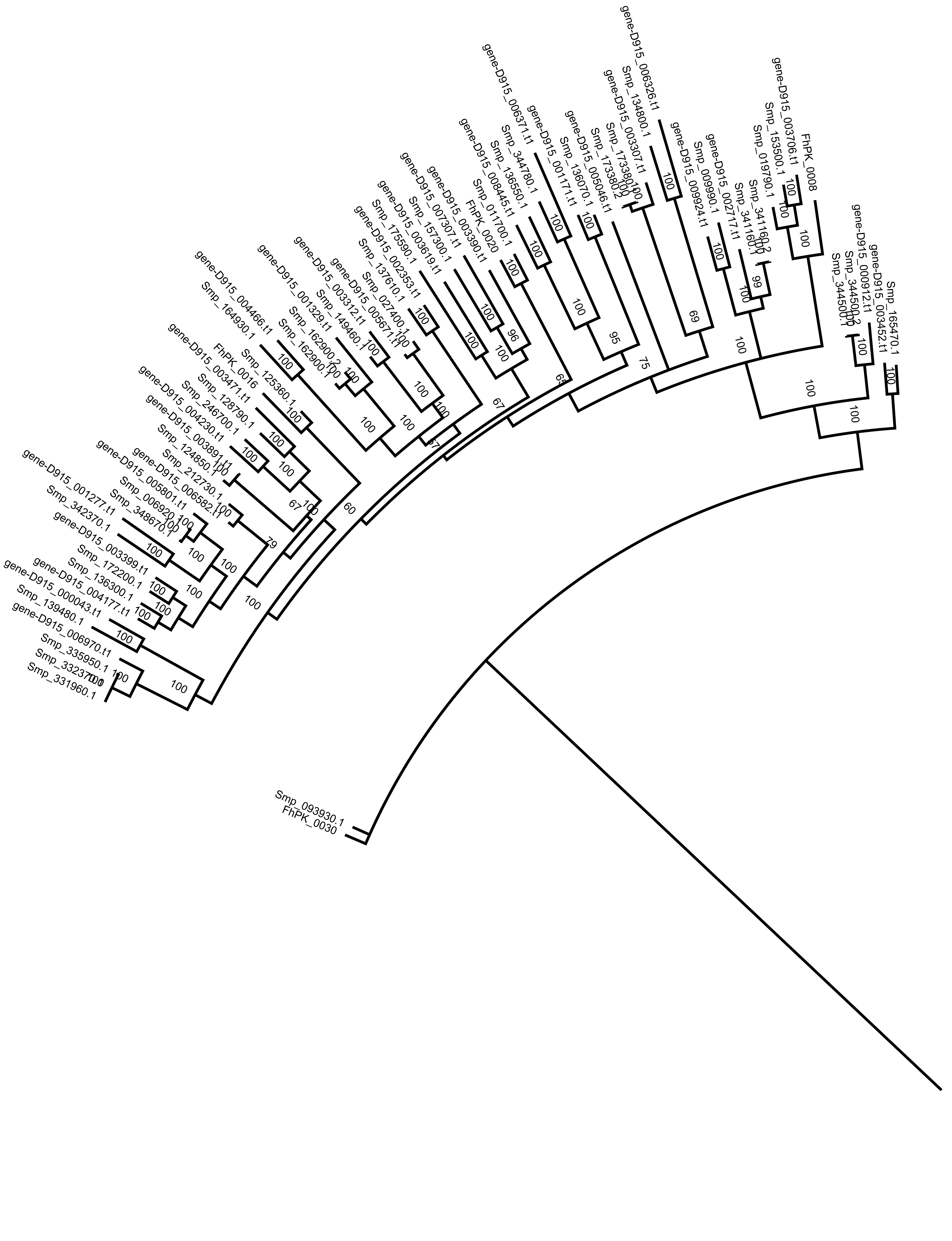

Supplementary Figure 10. TKL kinase group

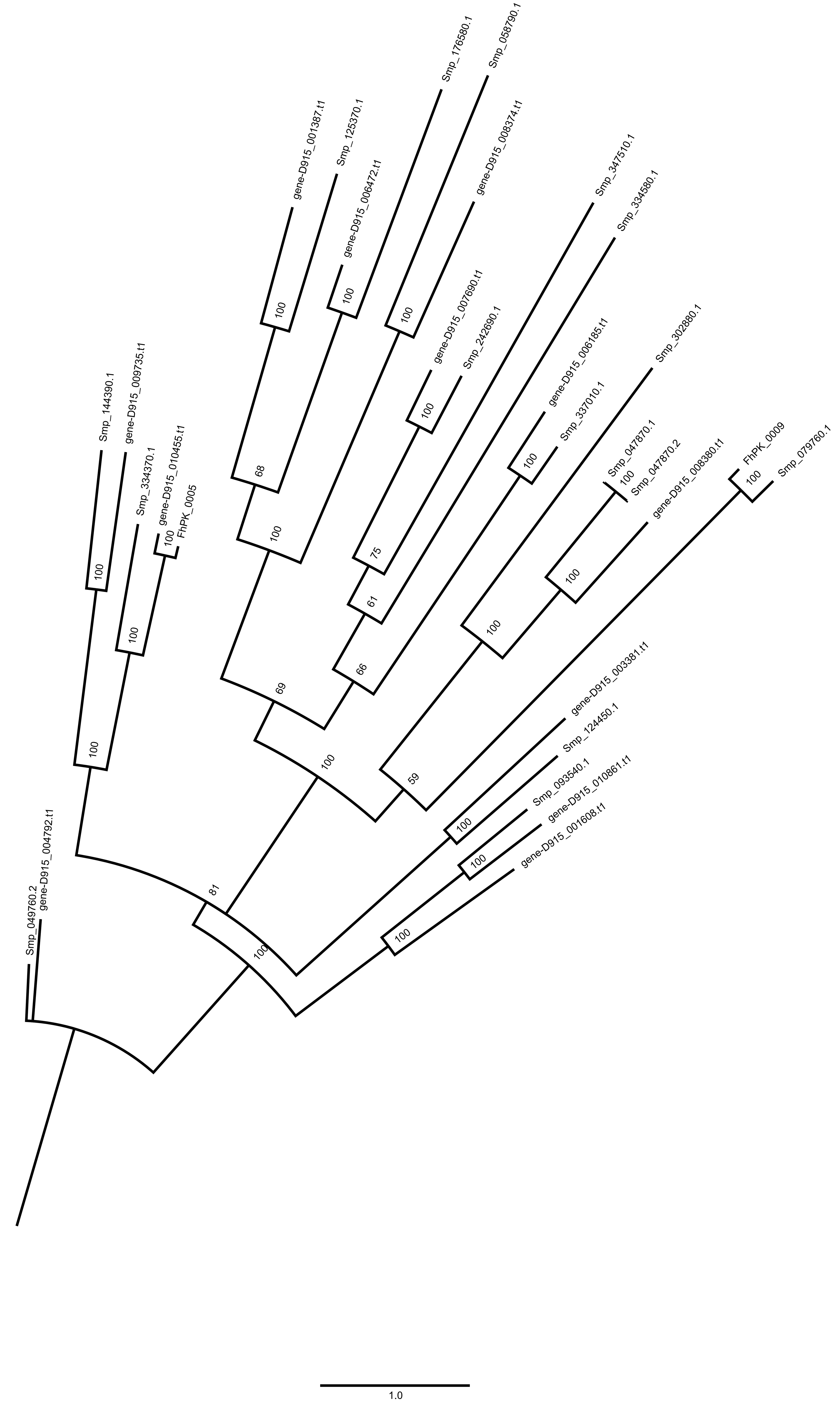

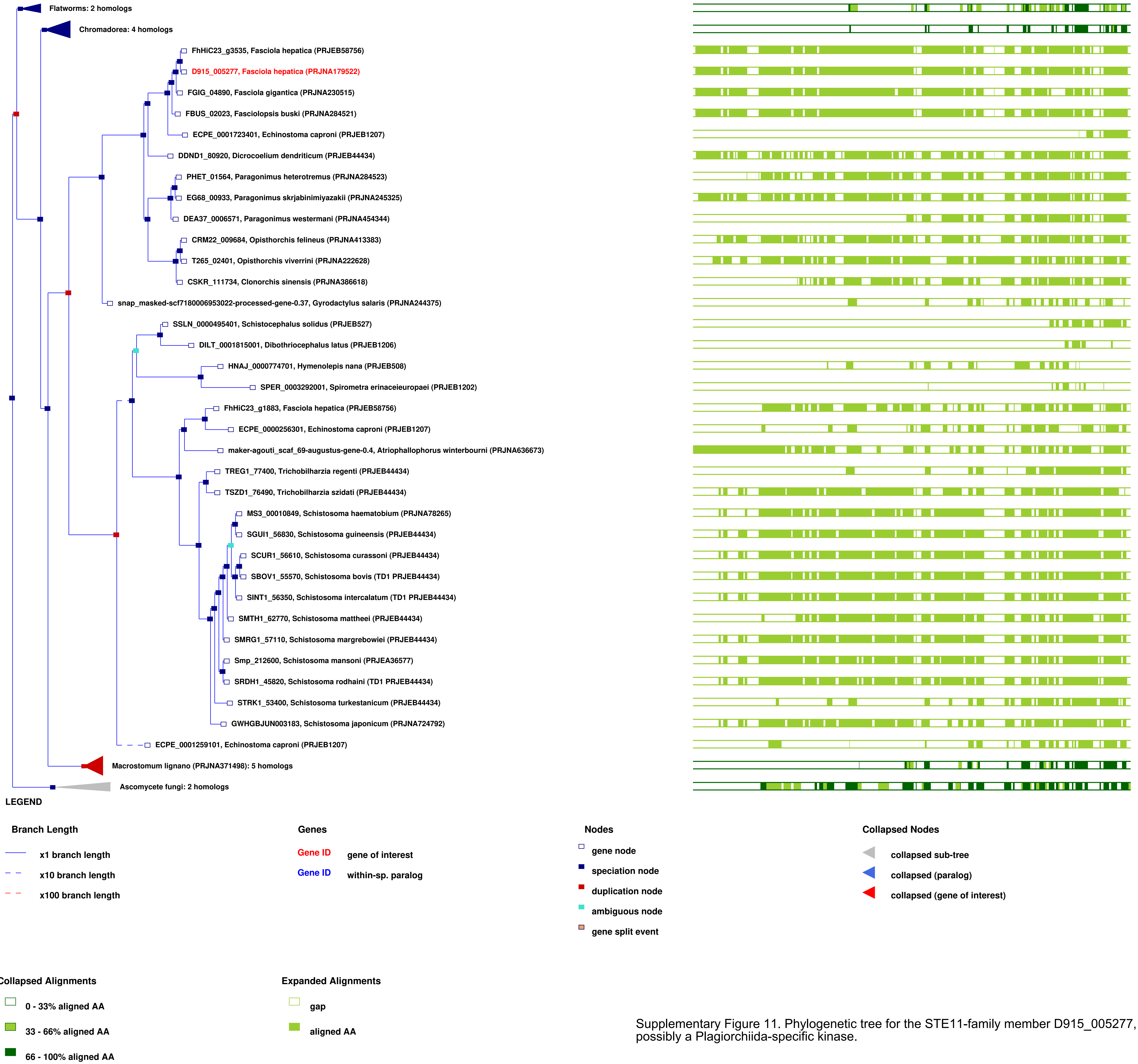

Supplementary Figure 11. Phylogenetic tree for the STE11-family member D915_005277, possibly a Plagiorchiida-specific kinase.

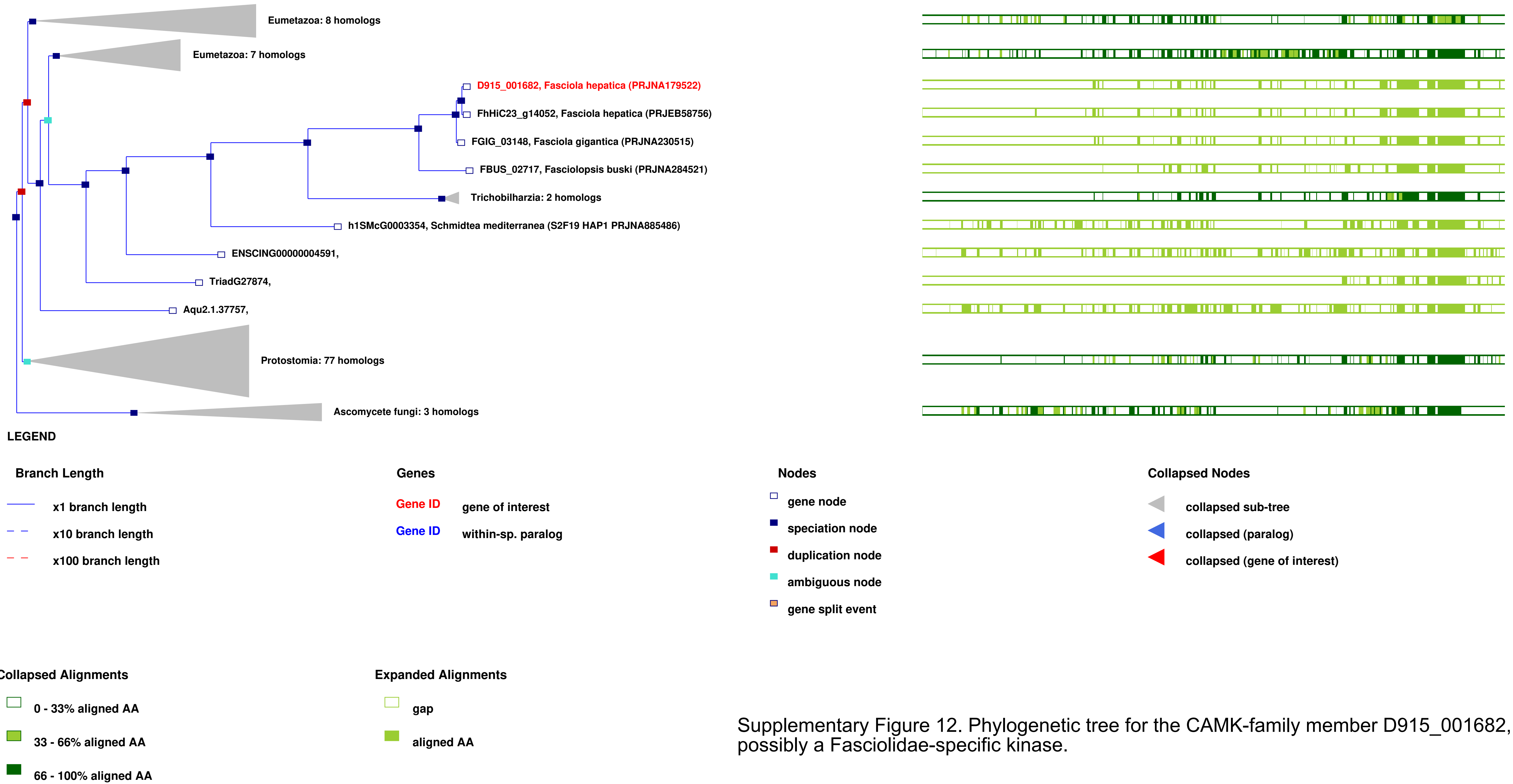

Supplementary Figure 12. Phylogenetic tree for the CAMK-family member D915_001682, possibly a Fasciolidae-specific kinase.

Supplementary data 2 | Table 1 | The statistical values for control vs vandetanib-treated adult and immature flukes. The mean motility score of flukes (n = 4) after 24, 48 and 72 h treatment for three independent experiments is listed (score 0 = dead, 1 = heavily reduced, 2= moderately reduced, 3 = normal movement). Wilcoxon rank sum test was performed to obtain the p value for each concentration vs control at different time points. The significance was assessed against control adult and immature flukes (n = 4), which exhibited the motility score of 3 (not shown). NaN = Not a Number, ns = not significant (p value ≥ 0.05), * = p value < 0.05

Time [h]	Stage	Concentration [μM]	Mean Score	SEM	p value	Statistical significance
24	Adult	25	3.00	0	NaN	NaN
24	Adult	50	2.25	0.25	0.060	ns
24	Adult	100	1.00	0	0.013	*
24	Immature	25	1.50	0.28	0.019	*
24	Immature	50	0.50	0.28	0.019	*
24	Immature	100	0	0	0.013	*
48	Adult	25	2.75	0.25	0.453	ns
48	Adult	50	1.75	0.47	0.066	ns
48	Adult	100	0	0	0.013	*
48	Immature	25	0.50	0.28	0.019	*
48	Immature	50	0	0	0.013	*
48	Immature	100	0	0	0.013	*
72	Adult	25	2.50	0.28	0.181	ns
72	Adult	50	1.50	0.64	0.068	ns
72	Adult	100	0	0	0.013	*
72	Immature	25	0.50	0.28	0.019	*
72	Immature	50	0	0	0.013	*
72	Immature	100	0	0	0.013	*

Supplementary data 2 | Table 2 | The statistical values for control vs foretinib-treated adult and immature flukes. The mean motility score of flukes (n = 4) after 24, 48 and 72 h treatment for three independent experiments is listed (score 0 = dead, 1 = heavily reduced, 2= moderately reduced, 3 = normal movement). Wilcoxon rank sum test was performed to obtain the p value for each concentration vs control at different time points. The significance was assessed against control adult and immature flukes (n = 4), which exhibited the motility score of 3 (not shown). NaN = Not a Number, ns = not significant (p value ≥ 0.05), * = p value < 0.05

Time [h]	Stage	Concentration [μM]	Mean Score	SEM	p value	Statistical significance
24	Adult	25	3.00	0	NaN	NaN
24	Adult	50	3.00	0	NaN	NaN
24	Adult	100	2.00	0	0.013	*
24	Immature	25	2.50	0.28	0.181	ns
24	Immature	50	1.50	0.28	0.019	*
24	Immature	100	0.50	0.28	0.019	*
48	Adult	25	2.75	0.25	0.453	ns
48	Adult	50	2.50	0.28	0.181	ns
48	Adult	100	1.00	0.40	0.02	*
48	Immature	25	1.00	0.57	0.019	*
48	Immature	50	0.25	0.25	0.017	*
48	Immature	100	0.25	0.25	0.017	*
72	Adult	25	2.75	0.25	0.453	ns
72	Adult	50	1.50	0.28	0.019	*
72	Adult	100	0.50	0.28	0.019	*
72	Immature	25	0.50	0.28	0.019	*
72	Immature	50	0	0	0.013	*
72	Immature	100	0	0	0.013	*

Supplementary data 2 | Table 3 | The statistical values for control vs tyrosine kinase-IN-1-treated adult and immature flukes. The mean motility score of flukes (n = 4) after 24, 48 and 72 h treatment for three independent experiments is listed (score 0 = dead, 1 = heavily reduced, 2= moderately reduced, 3 = normal movement). Wilcoxon rank sum test was performed to obtain the p value for each concentration vs control at different time points. The significance was assessed against control adult and immature flukes (n = 4), which exhibited the motility score of 3 (not shown). NaN = Not a Number, ns = not significant (p value ≥ 0.05), * = p value < 0.05

Time [h]	Stage	Concentration [μM]	Mean Score	SEM	p value	Statistical significance
24	Adult	25	3.00	0	NaN	NaN
24	Adult	50	3.00	0	NaN	NaN
24	Adult	100	0.50	0.28	0.019	*
24	Immature	25	2.00	0	0.013	*
24	Immature	50	0.50	0.28	0.019	*
24	Immature	100	0.25	0.25	0.017	*
48	Adult	25	3.00	0	NaN	NaN
48	Adult	50	2.50	0.28	0.181	ns
48	Adult	100	0	0	0.013	*
48	Immature	25	1.00	0	0.013	*
48	Immature	50	0	0	0.013	*
48	Immature	100	0.25	0.25	0.017	*
72	Adult	25	2.75	0.25	0.453	ns
72	Adult	50	2.00	0.40	0.066	ns
72	Adult	100	0	0	0.013	*
72	Immature	25	0.75	0.25	0.017	*
72	Immature	50	0	0	0.013	*
72	Immature	100	0	0	0.013	*

Supplementary data 2 | Table 4 | The statistical values for control vs ruboxistaurin-treated adult and immature flukes. The mean motility score of flukes (n = 4) after 24, 48 and 72 h treatment for three independent experiments is listed (score 0 = dead, 1 = heavily reduced, 2= moderately reduced, 3 = normal movement). Wilcoxon rank sum test was performed to obtain the p value for each concentration vs control at different time points. The significance was assessed against control adult and immature flukes (n = 4), which exhibited the motility score of 3 (not shown). ns = not significant (p value ≥ 0.05), * = p value < 0.05

Time [h]	Stage	Concentration [μM]	Mean Score	SEM	p value	Statistical significance
24	Adult	25	2.75	0.25	0.453	ns
24	Adult	50	1.00	0	0.013	*
24	Adult	100	0	0	0.013	*
24	Immature	25	1.50	0.28	0.019	*
24	Immature	50	0	0	0.013	*
24	Immature	100	0	0	0.013	*
48	Adult	25	2.50	0.28	0.181	ns
48	Adult	50	0.50	0.28	0.019	*
48	Adult	100	0	0	0.013	*
48	Immature	25	1.00	0.57	0.019	*
48	Immature	50	0	0	0.013	*
48	Immature	100	0	0	0.013	*
72	Adult	25	2.00	0	0.013	*
72	Adult	50	0	0	0.013	*
72	Adult	100	0	0	0.013	*
72	Immature	25	0.50	0.28	0.019	*
72	Immature	50	0	0	0.013	*
72	Immature	100	0	0	0.013	*

Supplementary data 2 | Table 5 | The statistical values for control vs triclabendazole-treated adult and immature flukes. The mean motility score of flukes (n = 4) after 24, 48 and 72 h treatment for three independent experiments is listed (score 0 = dead, 1 = heavily reduced, 2= moderately reduced, 3 = normal movement). Wilcoxon rank sum test was performed to obtain the p value for each concentration vs control at different time points. The significance was assessed against control adult and immature flukes (n = 4), which exhibited the motility score of 3 (no shown). ns = not significant (p value ≥ 0.05), * = p value < 0.05

Time [h]	Stage	Concentration [μM]	Mean Score	SEM	p value	Statistical significance
24	Adult	25	2.50	0.28	0.181	ns
24	Adult	50	2.00	0.40	0.066	ns
24	Adult	100	0.50	0.28	0.019	*
24	Immature	25	2.00	0	0.013	*
24	Immature	50	1.00	0	0.013	*
24	Immature	100	0	0	0.013	*
48	Adult	25	2.00	0.40	0.066	ns
48	Adult	50	1.50	0.28	0.019	*
48	Adult	100	0	0	0.013	*
48	Immature	25	2.00	0	0.013	*
48	Immature	50	1.00	0	0.013	*
48	Immature	100	0	0	0.013	*
72	Adult	25	1.50	0.28	0.019	*
72	Adult	50	1.00	0	0.013	*
72	Adult	100	0	0	0.013	*
72	Immature	25	1.00	0	0.013	*
72	Immature	50	0	0	0.013	*
72	Immature	100	0	0	0.013	*

Supplementary data 2 | Table 6 | The clinical trial status of protein kinase inhibitors used *in vitro* against immature and adult flukes.

PK inhibitor	Clinical trial status	Pharma-cological target	Known adverse effects	Administra-tion scheme (dosage)	Refe-rences
Vandetanib (ZD6474)	FDA approved (medullary thyroid cancer)	Multi-receptor tyrosine kinase	Hypertension, QTc interval prolongation and increased AST and ALT levels	≤ 300 mg/day for 56 days	(1–6)
Foretinib (XL 880)	Investigational phase I/II but discontinued/withdrawn	Multi-receptor tyrosine kinase	Hypertension and proteinuria	80 mg/day for 28 days	(7,8)
Tyrosine kinase-IN-1 (XL999)	Terminated after phase II	Multi-receptor tyrosine kinase	Cardiac toxicities	2.4 mg/kg intravenous as a four-hour infusion weekly for 8 weeks	(9,10)
Ruboxistaurin (LY333531)	Investigational phase II/III (diabetic retinopathy)	PKCβ	Nasopharyngitis	32 mg/day for two years	(11–13)

References:

1. Herbst RS, Heymach J V., O'Reilly MS, Onn A, Ryan AJ. Vandetanib (ZD6474): An orally available receptor tyrosine kinase inhibitor that selectively targets pathways critical for tumor growth and angiogenesis. Expert Opin Investig Drugs. 2007 Feb;16(2):239–49.
2. Lee SH, Lee JK, Ahn MJ, Kim DW, Sun JM, Keam B, et al. Vandetanib in pretreated patients with advanced non-small cell lung cancer-harboring RET rearrangement: A phase II clinical trial. Annals of Oncology. 2017 Feb 1;28(2):292–7.
3. Inoue K, Torimura T, Nakamura T, Iwamoto H, Masuda H, Abe M, et al. Vandetanib, an inhibitor of VEGF receptor-2 and EGF receptor, suppresses tumor development and improves prognosis of liver cancer in mice. Clinical Cancer Research. 2012 Jul 15;18(14):3924–33.
4. FDA. FDA. 2011 [cited 2025 Oct 30]. FDA approves new treatment for rare form of thyroid cancer. Available from: https://web.archive.org/web/20120302105514/http://www.fda.gov/NewsEvents/Newsroom/PressAnnouncements/2011/ucm250168.htm
5. Deshpande H, Roman S, Thumar J, Sosa JA. Vandetanib (ZD6474) in the treatment of medullary thyroid cancer. Clin Med Insights Oncol. 2011 Jan;5(CMO-S6197):213–21.
6. Michael Vozniak J, Jacobs JM. Vandetanib. J Adv Pract Oncol. 2012 Mar 1;3(2):112–6.
7. Shapiro GI, McCallum S, Adams LM, Sherman L, Weller S, Swann S, et al. A Phase 1 dose-escalation study of the safety and pharmacokinetics of once-daily oral foretinib, a multi-kinase inhibitor, in patients with solid tumors. Invest New Drugs. 2013 Jun;31(3):742–50.
8. GlaxoSmithKline. www.clinicaltrials.gov. 2016 [cited 2025 Oct 30]. A phase II study to evaluate Foretinib in genomic subpopulations of subjects with non-small-cell lung cancer (NSCLC). Available from: https://clinicaltrials.gov/study/NCT02034097

9. Symphony Evolution Inc. www.clinicaltrials.gov. 2007 [cited 2025 Oct 30]. A phase 2 study of XL999 administered intravenously to subjects with metastatic colorectal cancer. Available from: https://clinicaltrials.gov/study/NCT00277303
10. Symphony Evolution Inc. www.clinicaltrials.gov. 2008 [cited 2025 Oct 30]. A phase 2 study of XL999 administered intravenously to subjects with non-small-cell lung cancer. Available from: https://clinicaltrials.gov/study/NCT00277329
11. Javey G, Schwartz SG, Flynn HW, Aiello LP, Sheetz MJ. Ruboxistaurin: Review of safety and efficacy in the treatment of diabetic retinopathy. Clin Med Insights Ther. 2010 Jan;2:CMS5046.
12. Sheetz MJ, Aiello LP, Davis MD, Danis R, Bek T, Cunha-Vaz J, et al. The effect of the oral PKCβ inhibitor ruboxistaurin on vision loss in two phase 3 studies. Invest Ophthalmol Vis Sci. 2013 Mar 1;54(3):1750–7.
13. McGill JB, King GL, Berg PH, Price KL, Kles KA, Bastyr EJ, et al. Clinical safety of the selective PKC-β inhibitor, ruboxistaurin. Expert Opin Drug Saf. 2006 Nov 1;5(6):835–45.
